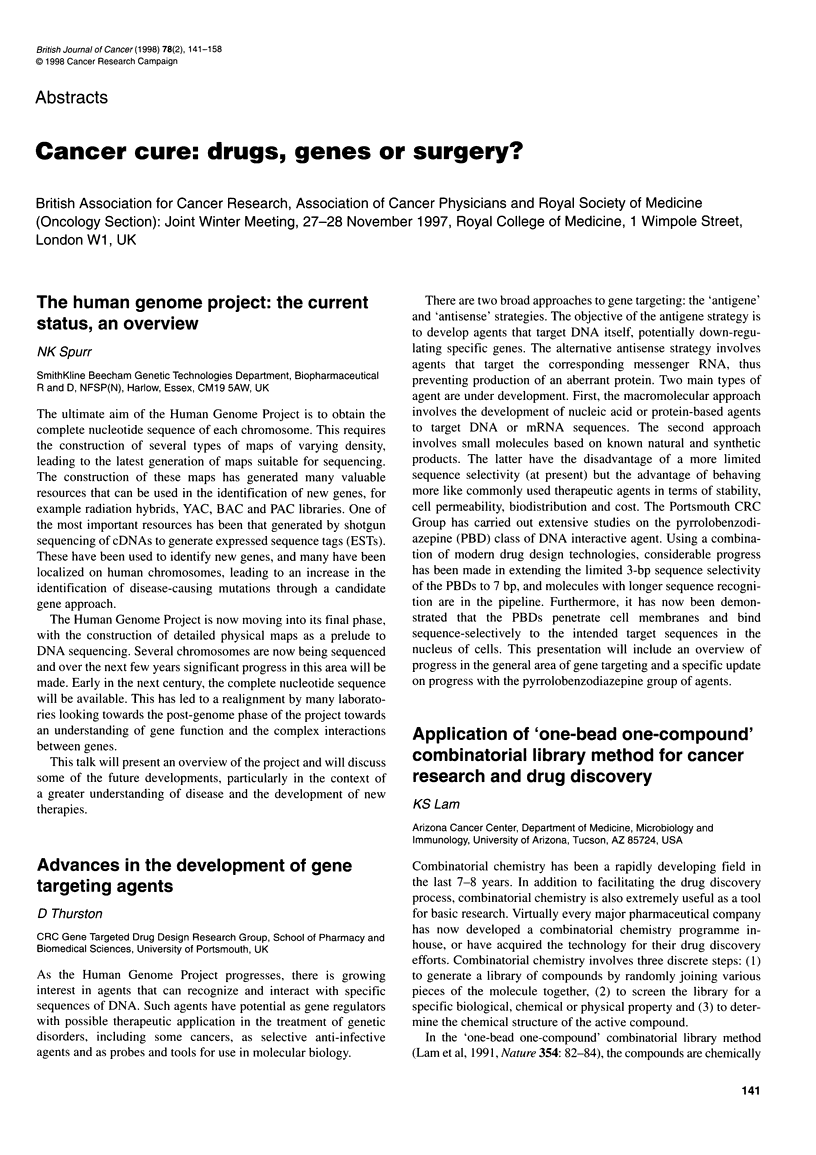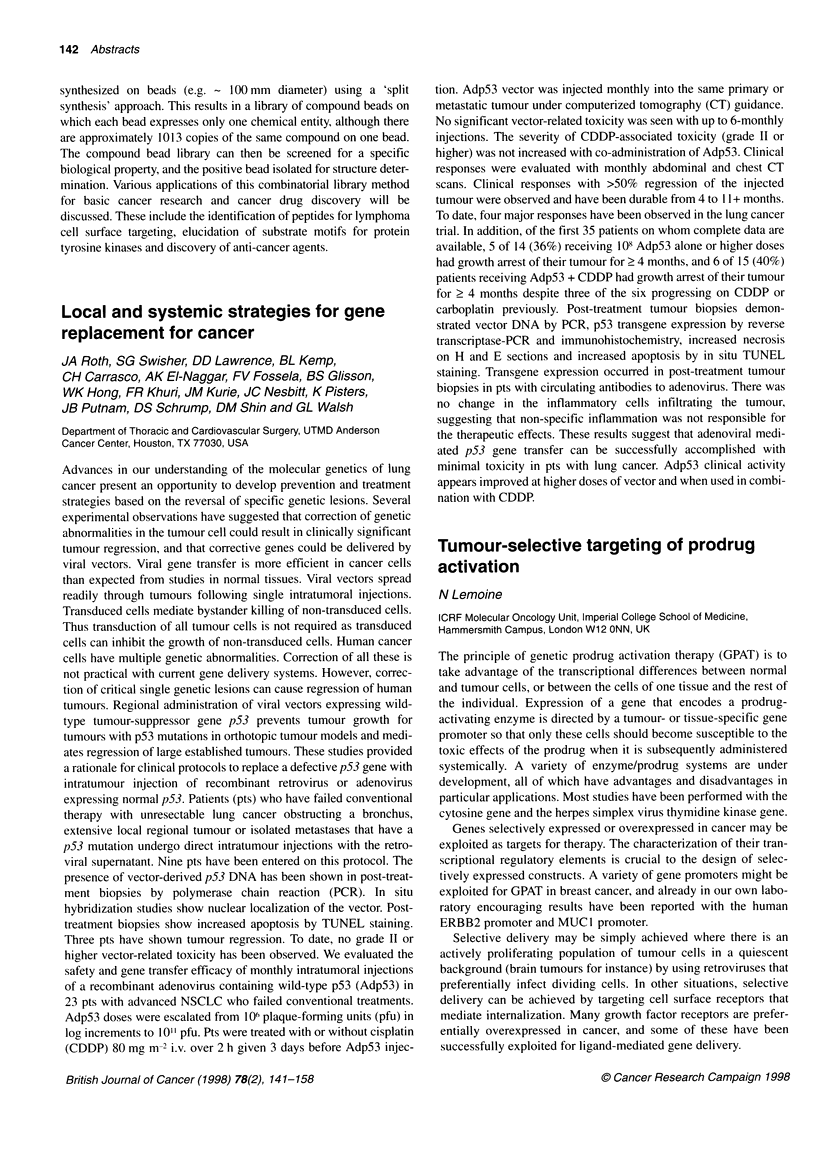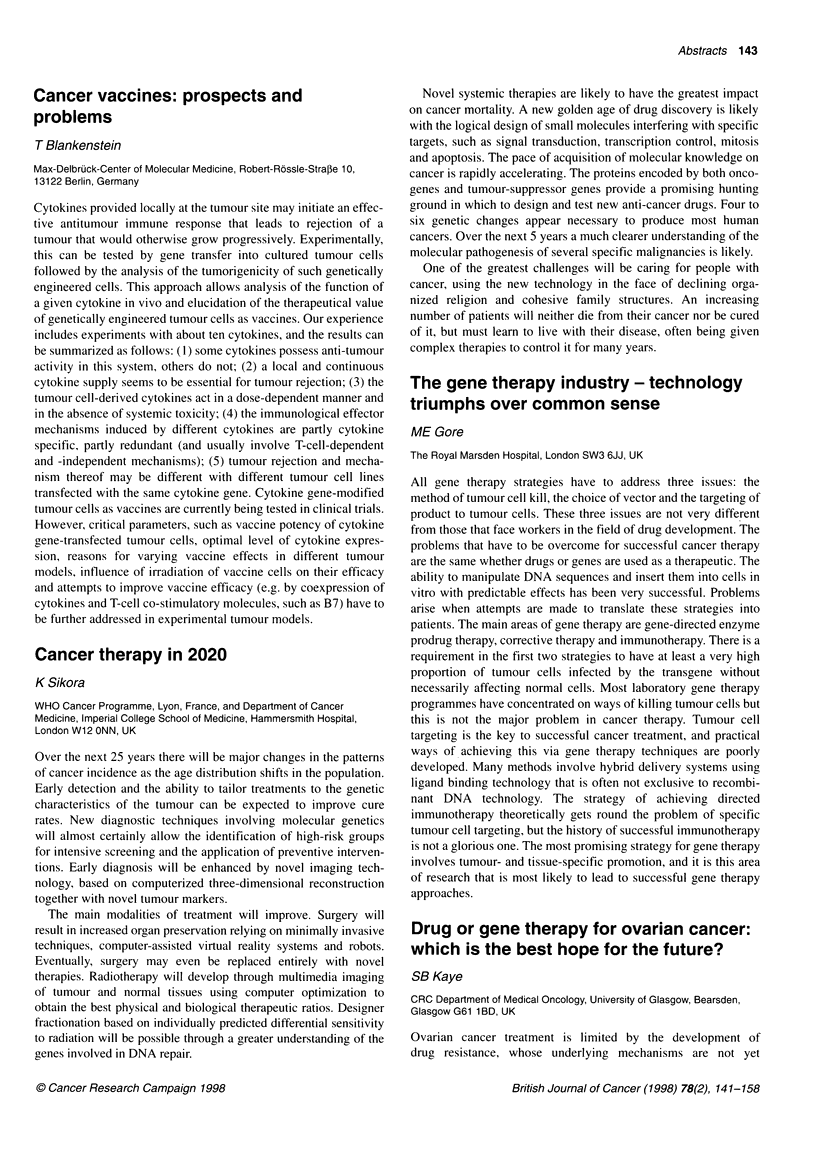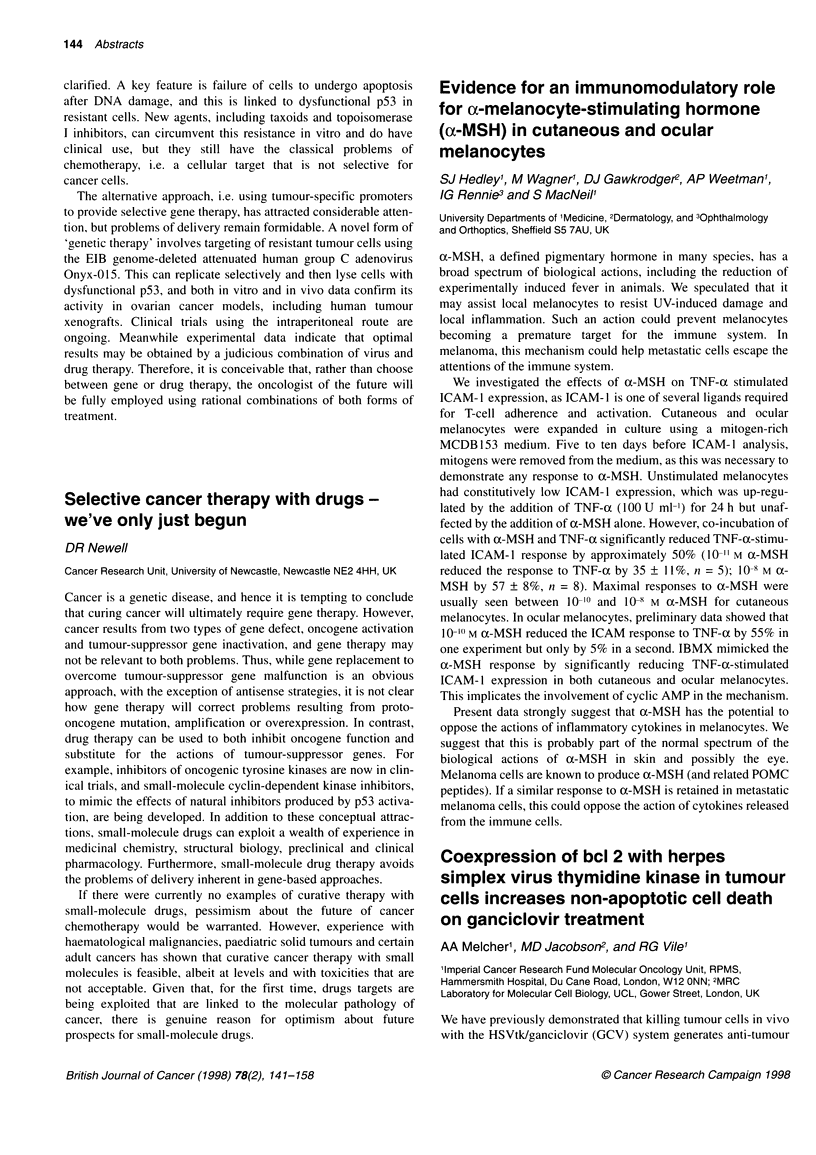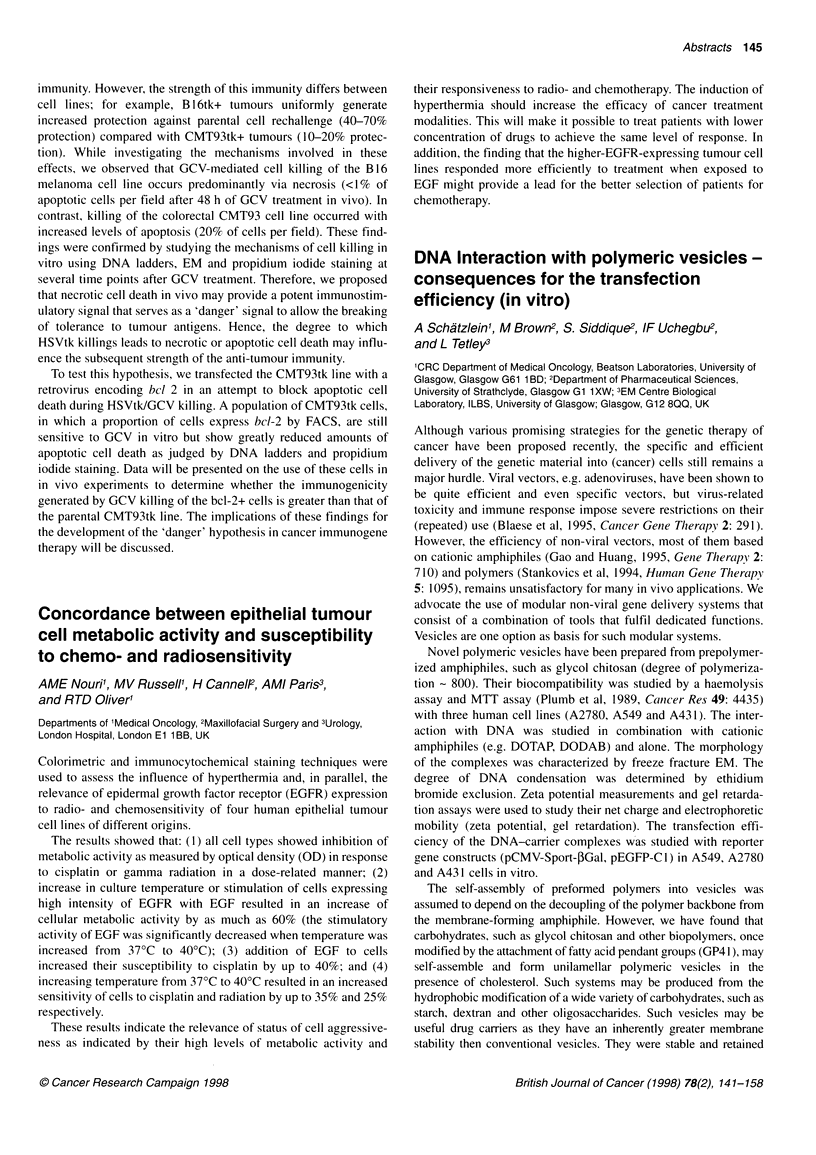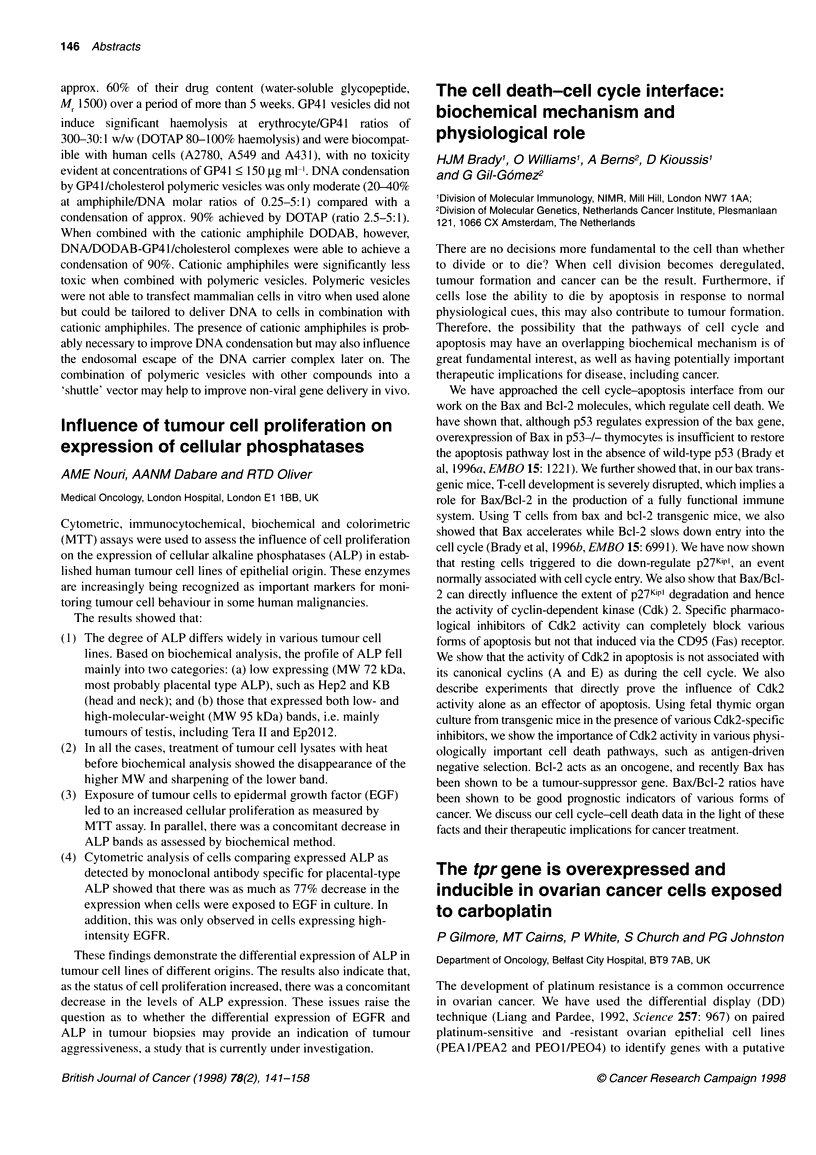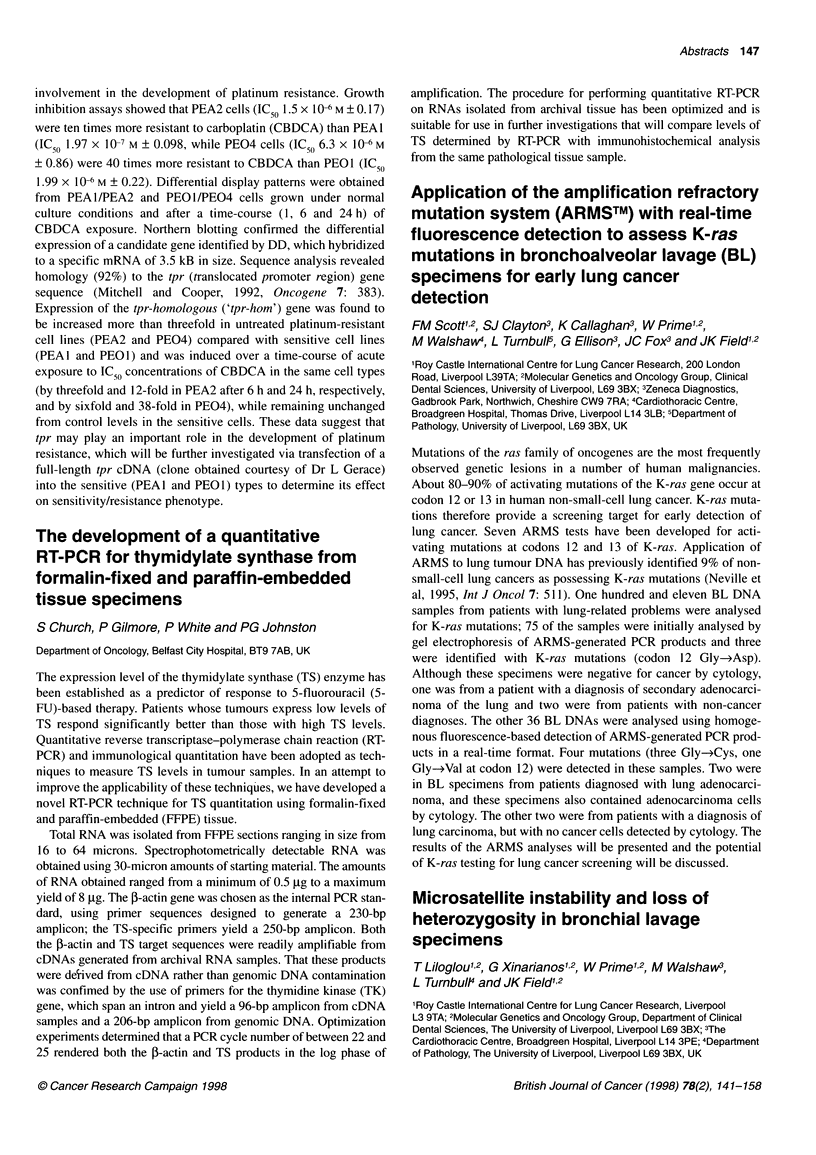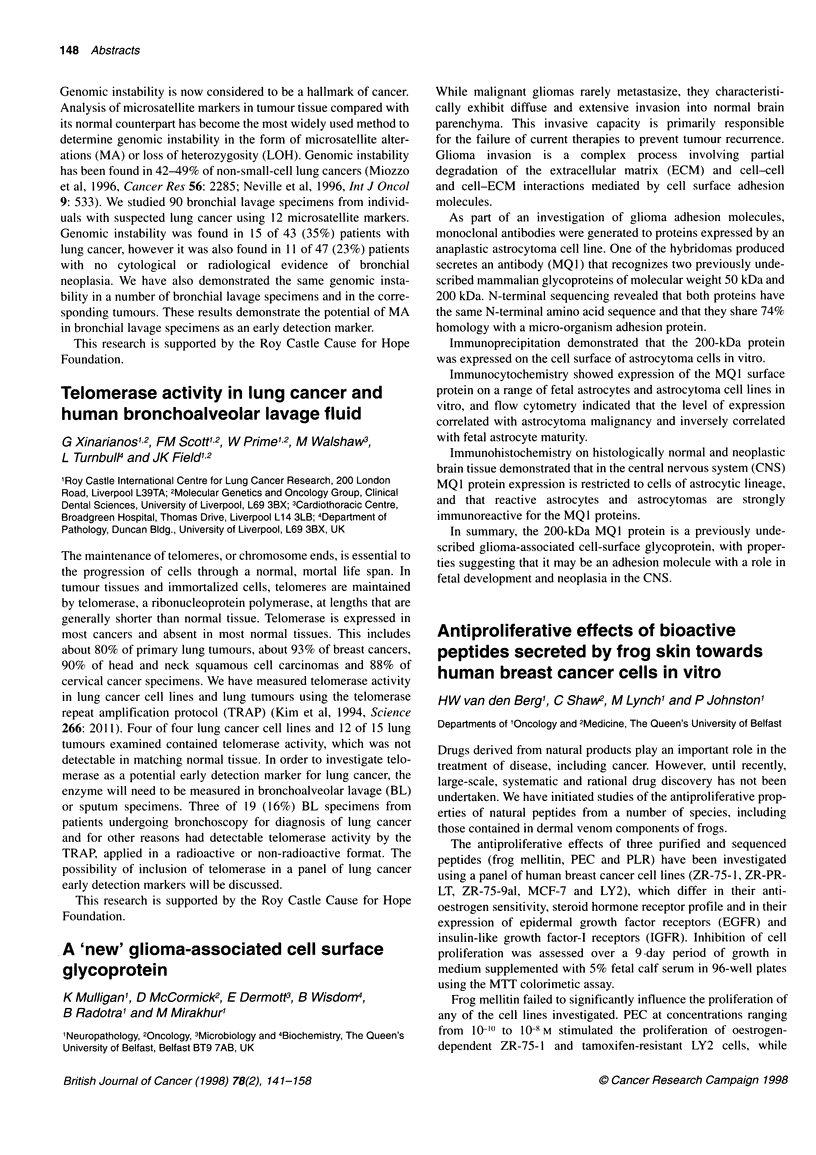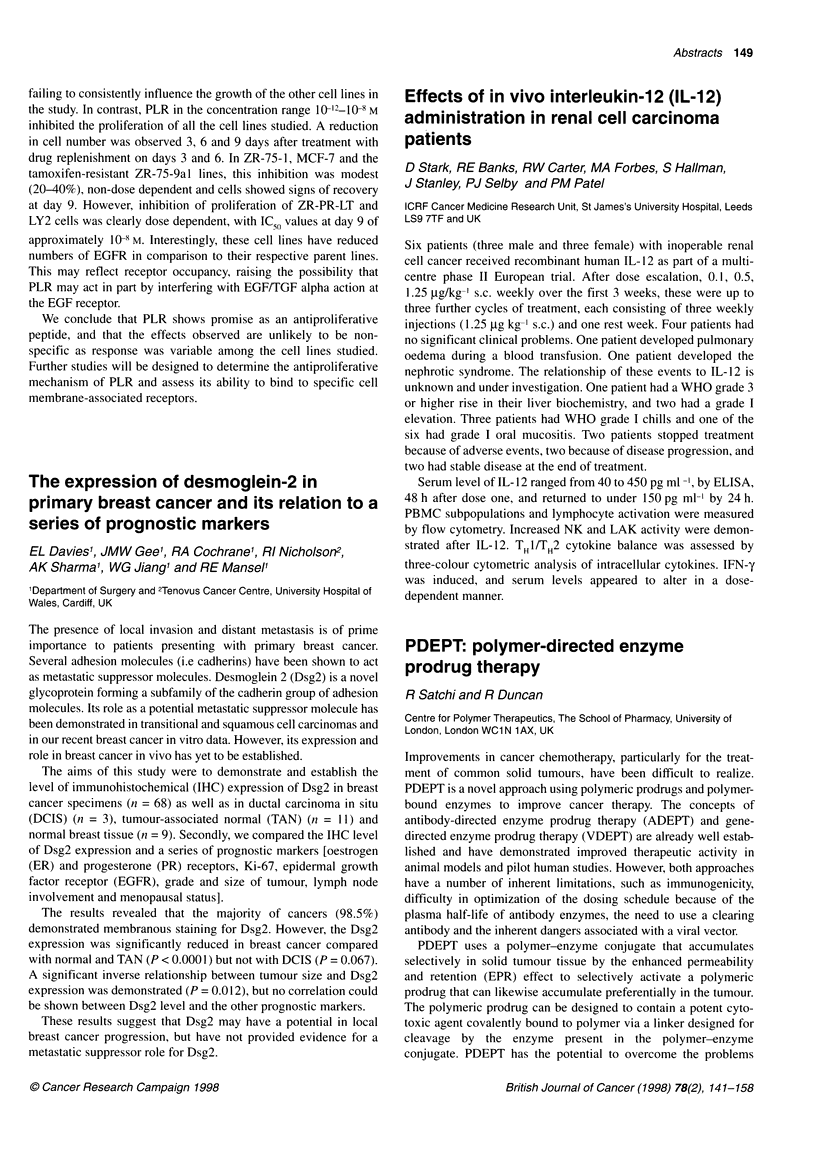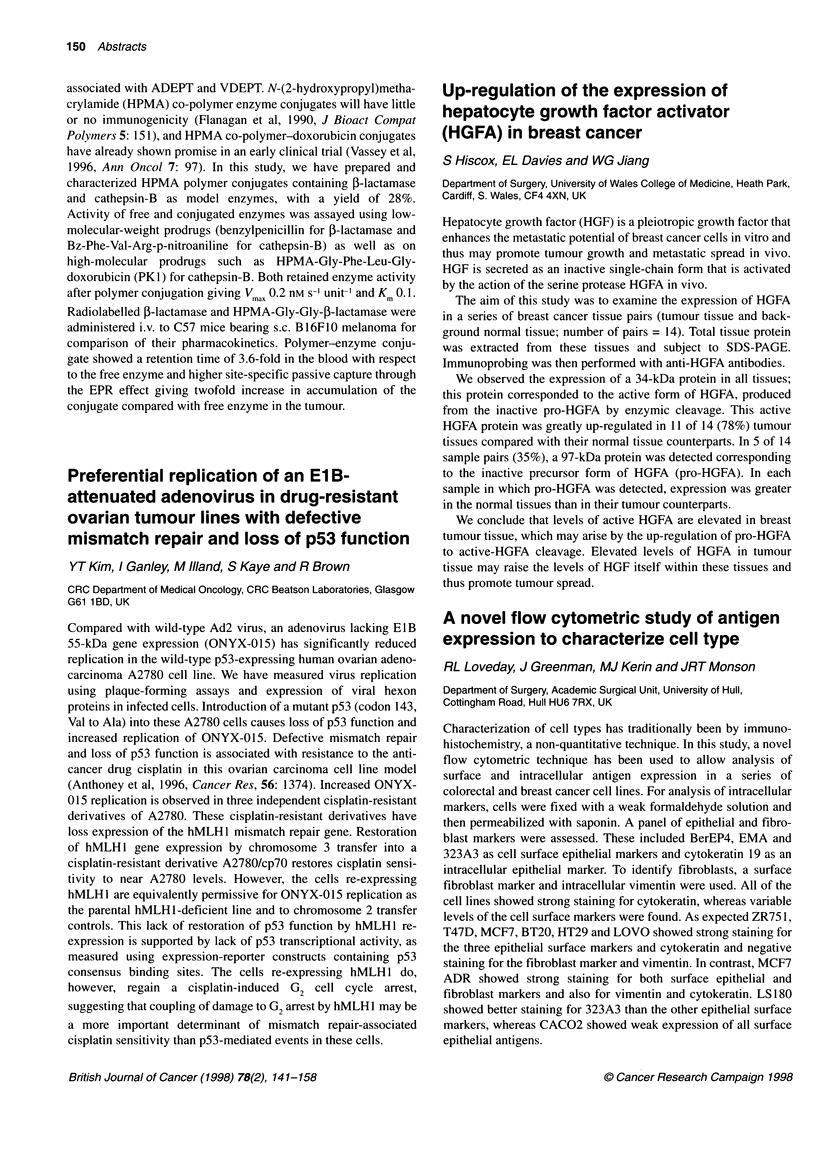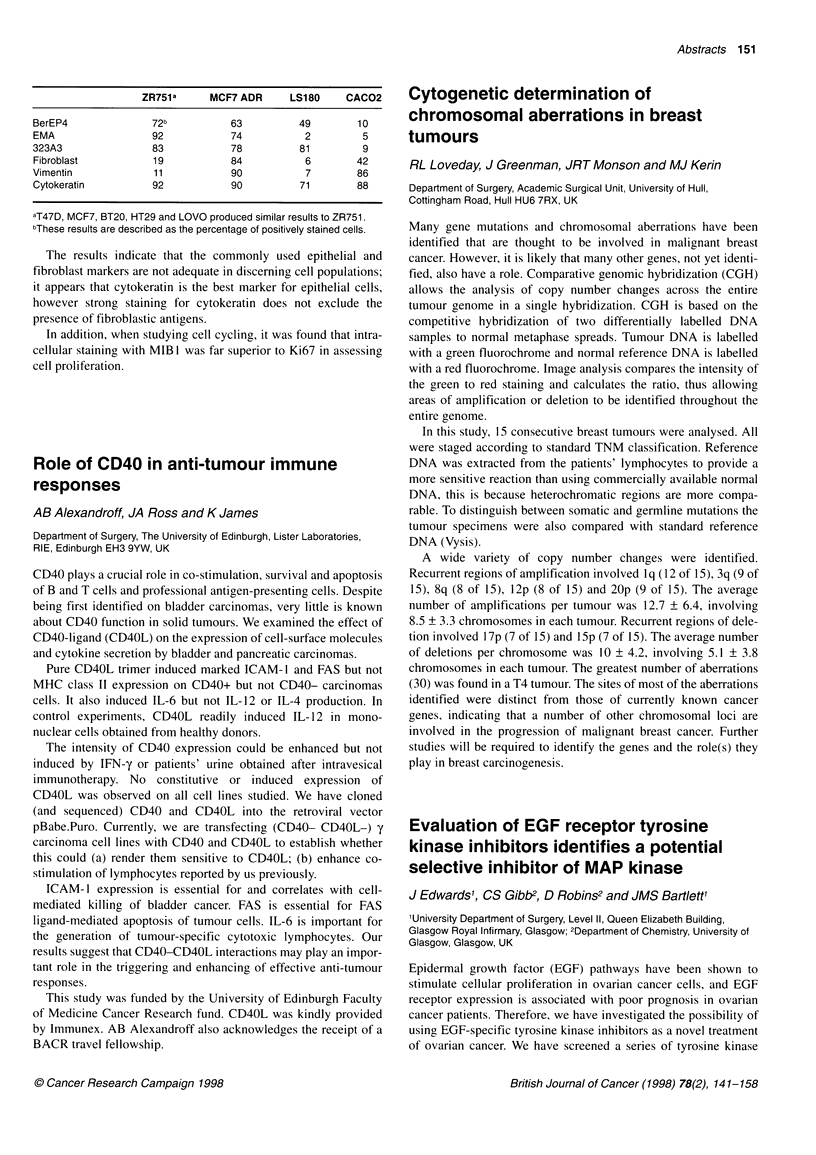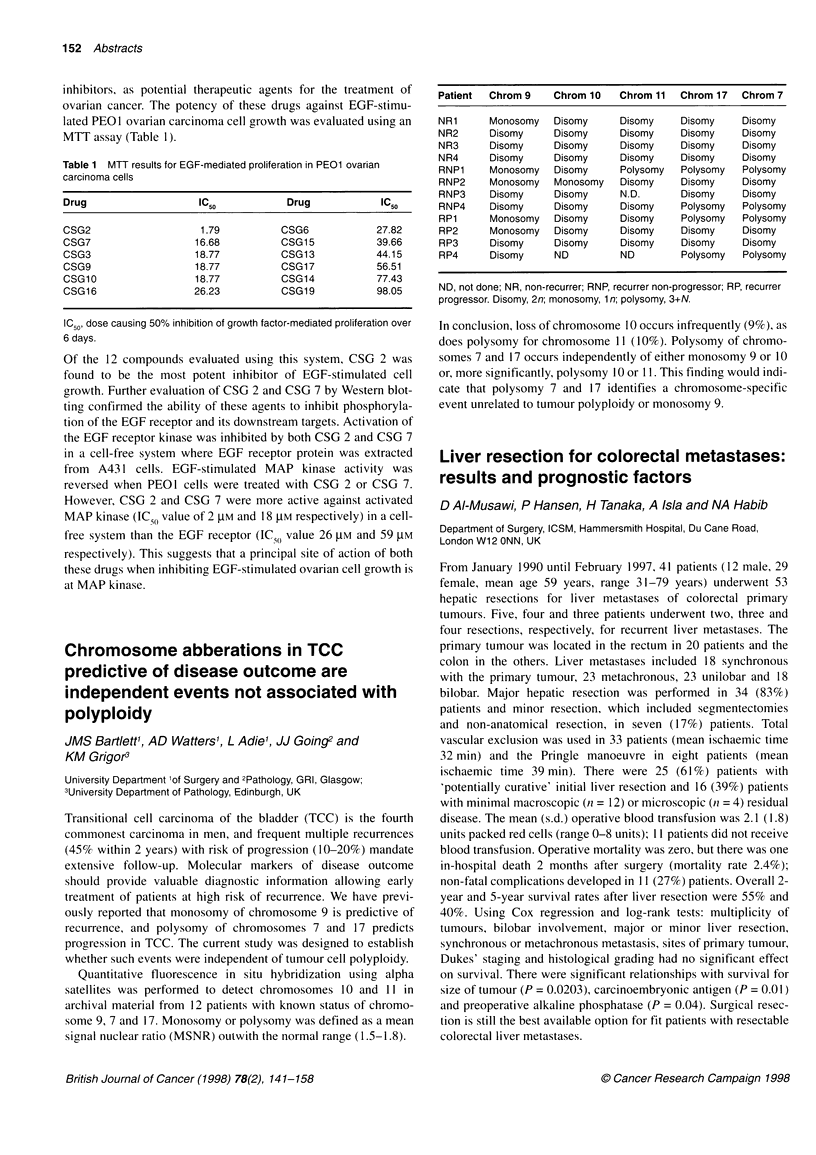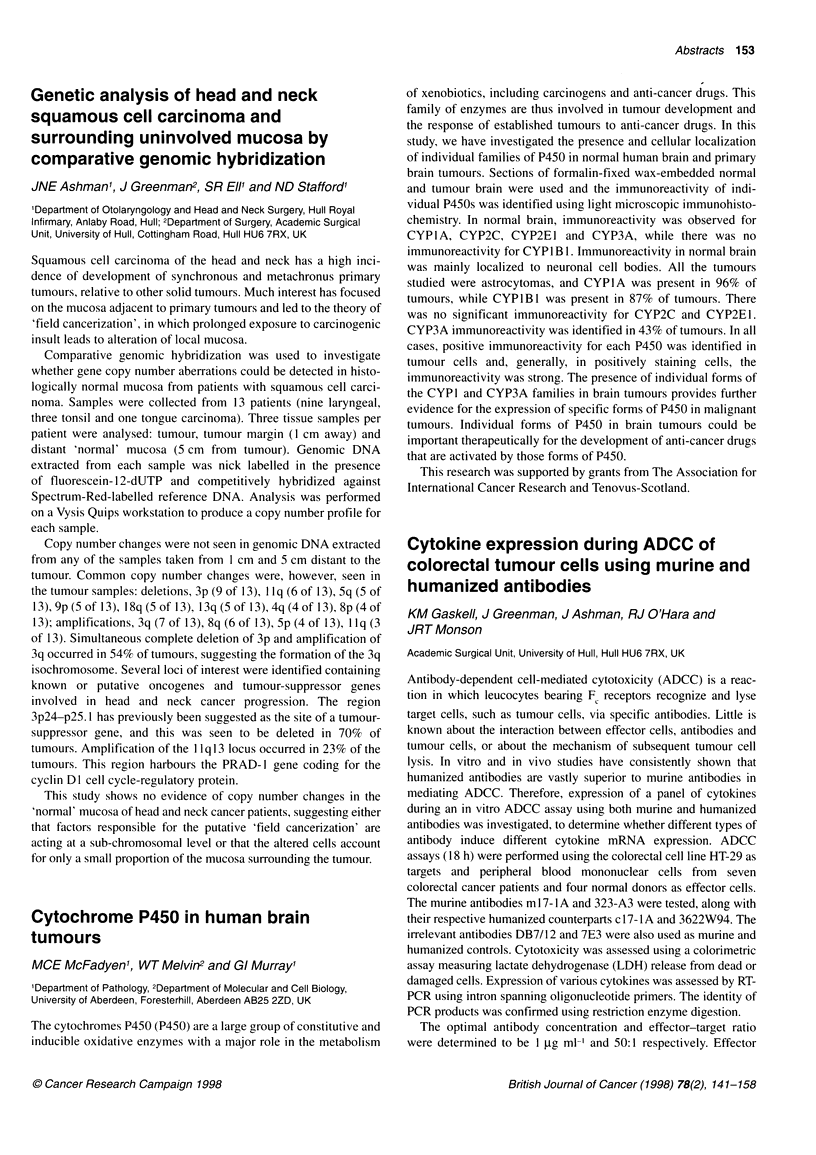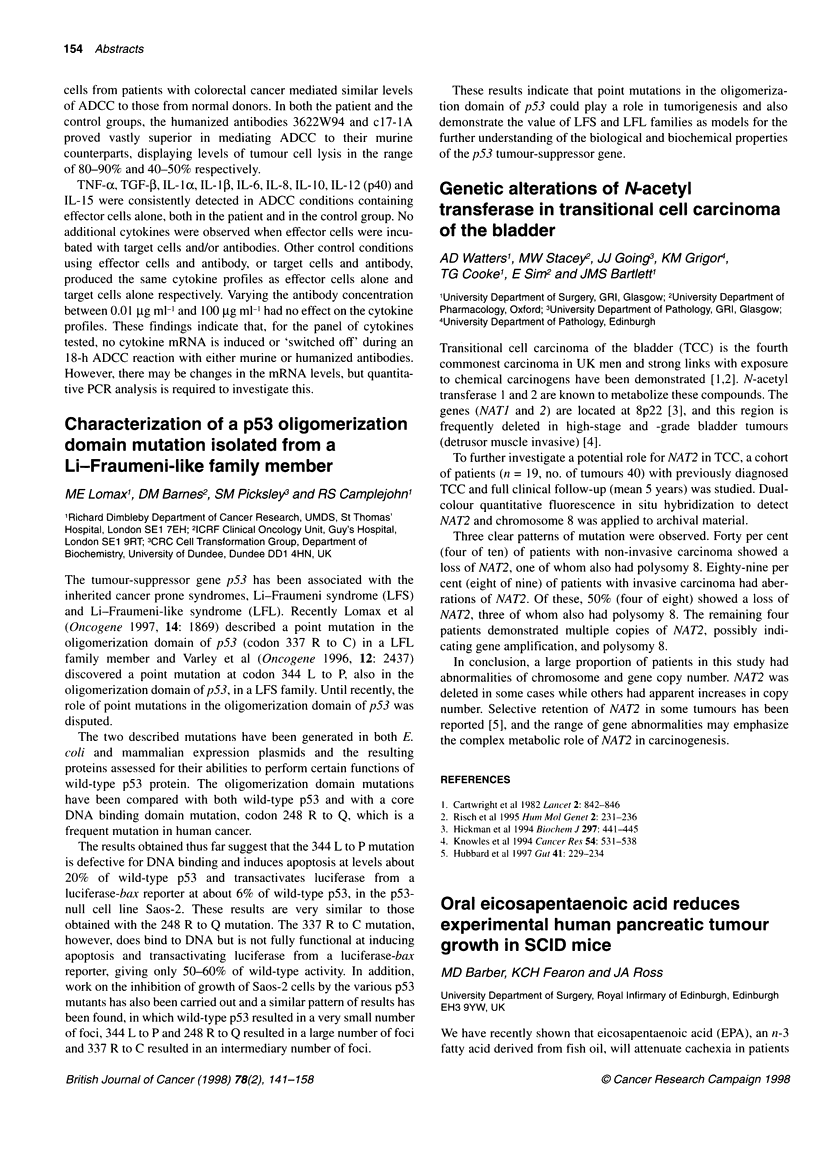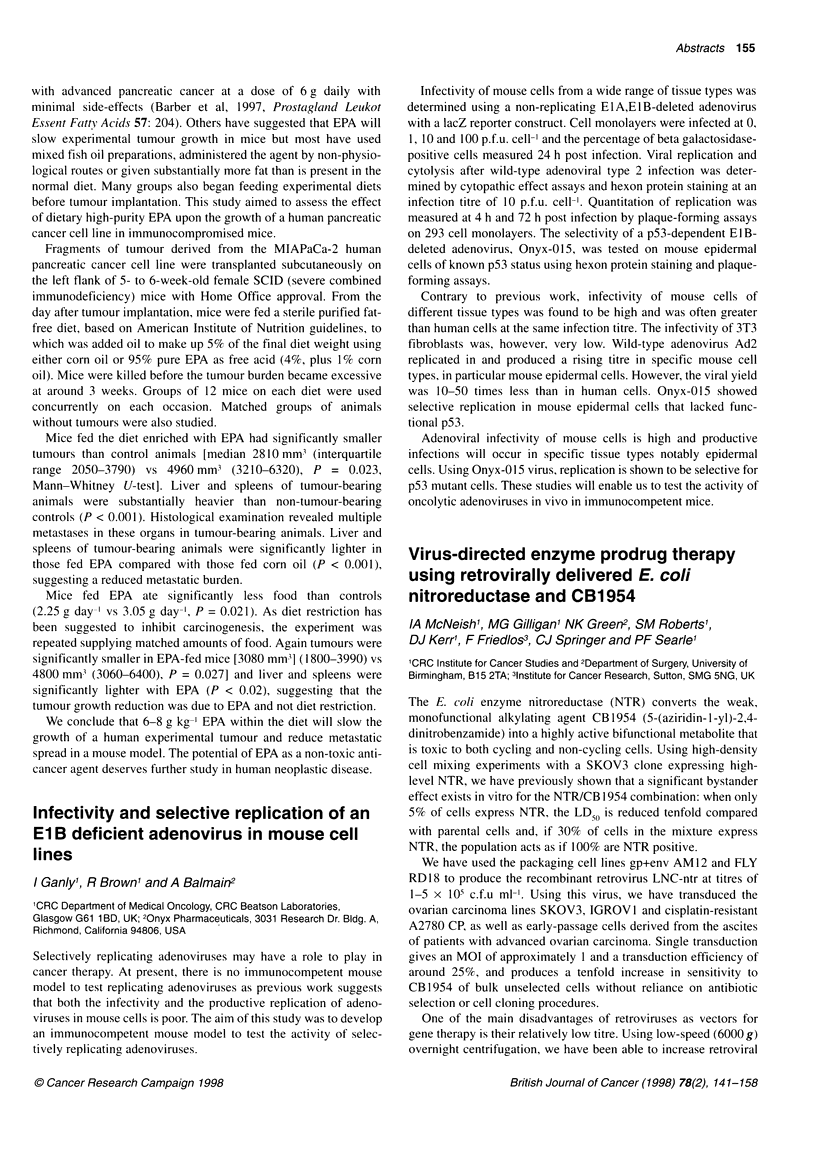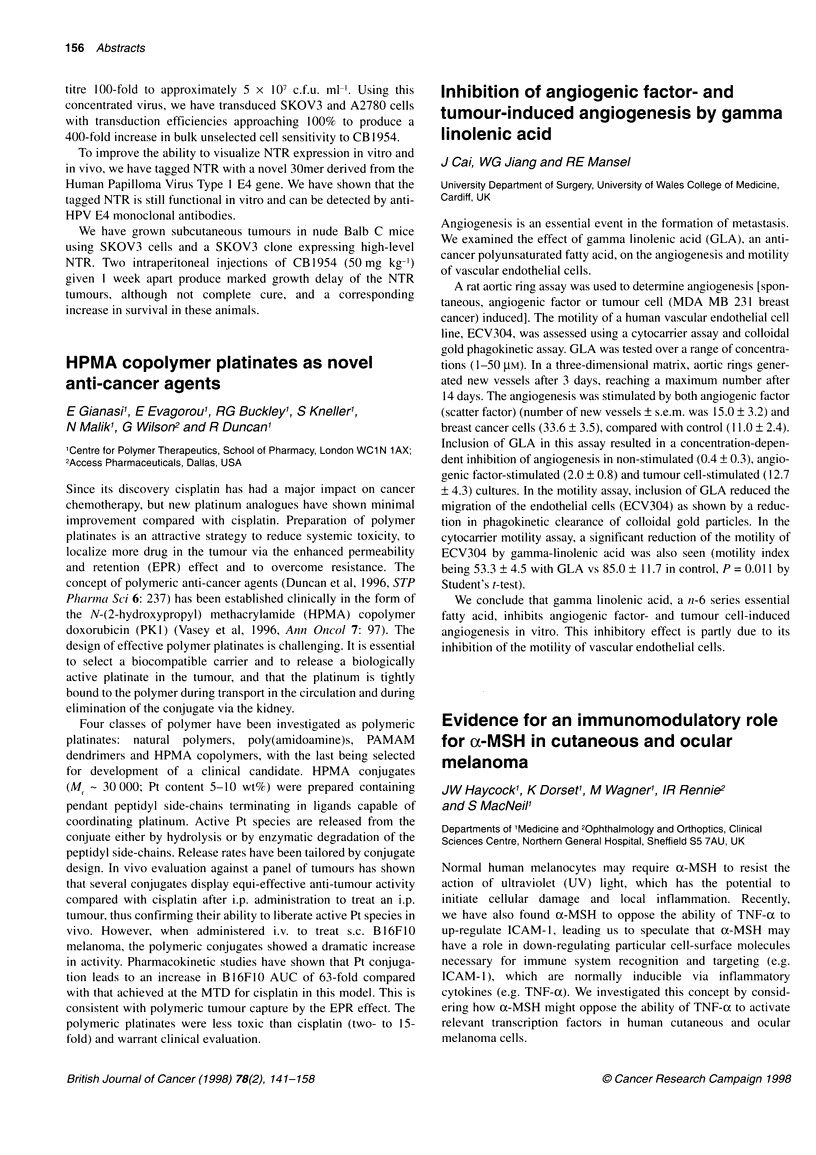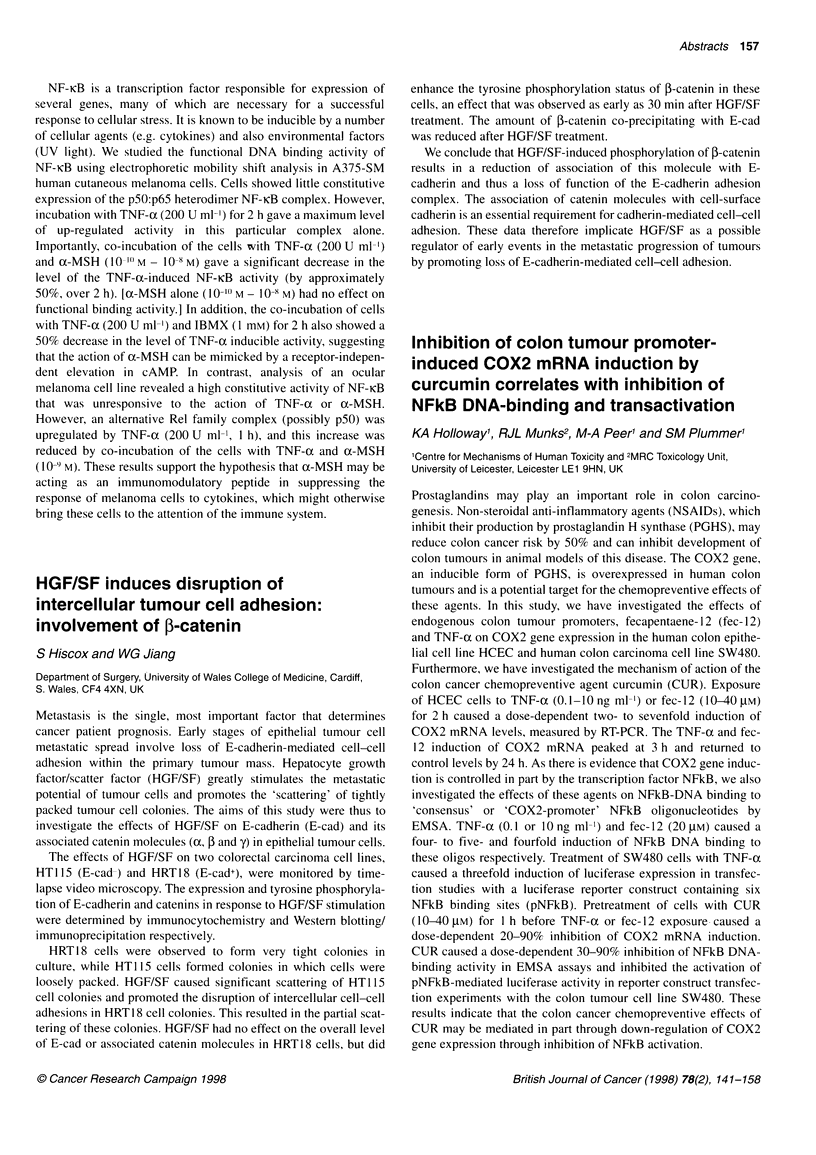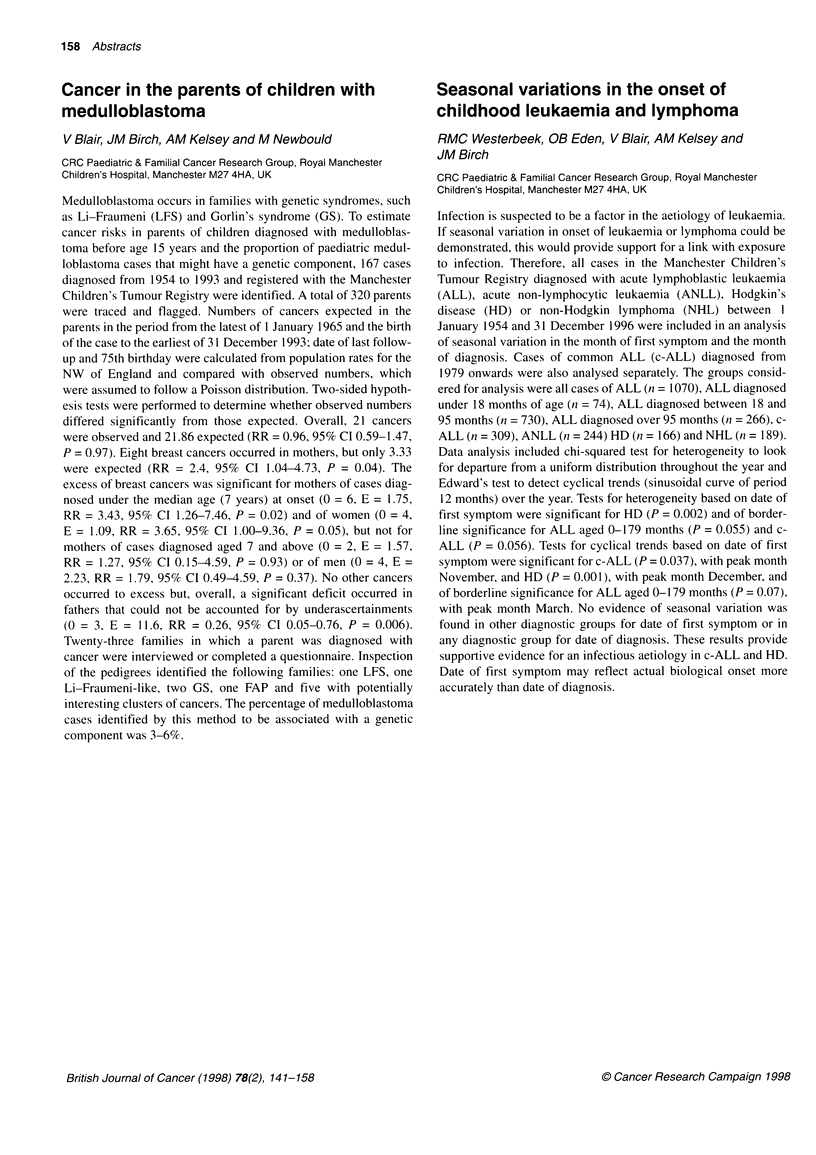# Cancer cure: drugs, genes or surgery?

**Published:** 1998-07

**Authors:** 


British Journal of Cancer (1998) 78(2), 141–158
© 1998 Cancer Research Campaign

Abstracts

### Cancer cure: drugs, genes or surgery?

British Association for Cancer Research, Association of Cancer Physicians and Royal Society of Medicine (Oncology Section): Joint Winter Meeting, 27–28 November 1997, Royal College of Medicine, 1 Wimpole Street, London W1, UK

### The human genome project: the current status, an overview

*NK Spurr*

SmithKline Beecham Genetic Technologies Department, Biopharmaceutical R and D, NFSP(N), Harlow, Essex, CM19 5AW, UK

The ultimate aim of the Human Genome Project is to obtain the complete nucleotide sequence of each chromosome. This requires the construction of several types of maps of varying density, leading to the latest generation of maps suitable for sequencing. The construction of these maps has generated many valuable resources that can be used in the identification of new genes, for example radiation hybrids, YAC, BAC and PAC libraries. One of the most important resources has been that generated by shotgun sequencing of cDNAs to generate expressed sequence tags (ESTs). These have been used to identify new genes, and many have been localized on human chromosomes, leading to an increase in the identification of disease-causing mutations through a candidate gene approach.

The Human Genome Project is now moving into its final phase, with the construction of detailed physical maps as a prelude to DNA sequencing. Several chromosomes are now being sequenced and over the next few years significant progress in this area will be made. Early in the next century, the complete nucleotide sequence will be available. This has led to a realignment by many laboratories looking towards the post-genome phase of the project towards an understanding of gene function and the complex interactions between genes.

This talk will present an overview of the project and will discuss some of the future developments, particularly in the context of a greater understanding of disease and the development of new therapies.

### Advances in the development of gene targeting agents

*D Thurston*

CRC Gene Targeted Drug Design Research Group, School of Pharmacy and Biomedical Sciences, University of Portsmouth, UK

As the Human Genome Project progresses, there is growing interest in agents that can recognize and interact with specific sequences of DNA. Such agents have potential as gene regulators with possible therapeutic application in the treatment of genetic disorders, including some cancers, as selective anti-infective agents and as probes and tools for use in molecular biology.

There are two broad approaches to gene targeting: the 'antigene' and 'antisense' strategies. The objective of the antigene strategy is to develop agents that target DNA itself, potentially down-regulating specific genes. The alternative antisense strategy involves agents that target the corresponding messenger RNA, thus preventing production of an aberrant protein. Two main types of agent are under development. First, the macromolecular approach involves the development of nucleic acid or protein-based agents to target DNA or mRNA sequences. The second approach involves small molecules based on known natural and synthetic products. The latter have the disadvantage of a more limited sequence selectivity (at present) but the advantage of behaving more like commonly used therapeutic agents in terms of stability, cell permeability, biodistribution and cost. The Portsmouth CRC Group has carried out extensive studies on the pyrrolobenzodiazepine (PBD) class of DNA interactive agent. Using a combination of modern drug design technologies, considerable progress has been made in extending the limited 3-bp sequence selectivity of the PBDs to 7 bp, and molecules with longer sequence recognition are in the pipeline. Furthermore, it has now been demonstrated that the PBDs penetrate cell membranes and bind sequence-selectively to the intended target sequences in the nucleus of cells. This presentation will include an overview of progress in the general area of gene targeting and a specific update on progress with the pyrrolobenzodiazepine group of agents.

### Application of 'one-bead one-compound' combinatorial library method for cancer research and drug discovery

*KS Lam*

Arizona Cancer Center, Department of Medicine, Microbiology and Immunology, University of Arizona, Tucson, AZ 85724, USA

Combinatorial chemistry has been a rapidly developing field in the last 7–8 years. In addition to facilitating the drug discovery process, combinatorial chemistry is also extremely useful as a tool for basic research. Virtually every major pharmaceutical company has now developed a combinatorial chemistry programme in-house, or have acquired the technology for their drug discovery efforts. Combinatorial chemistry involves three discrete steps: (1) to generate a library of compounds by randomly joining various pieces of the molecule together, (2) to screen the library for a specific biological, chemical or physical property and (3) to determine the chemical structure of the active compound.

In the 'one-bead one-compound' combinatorial library method (Lam et al, 1991, *Nature* **354**: 82–84), the compounds are chemically synthesized on beads (e.g. ~ 100 mm diameter) using a 'split synthesis' approach. This results in a library of compound beads on which each bead expresses only one chemical entity, although there are approximately 1013 copies of the same compound on one bead. The compound bead library can then be screened for a specific biological property, and the positive bead isolated for structure determination. Various applications of this combinatorial library method for basic cancer research and cancer drug discovery will be discussed. These include the identification of peptides for lymphoma cell surface targeting, elucidation of substrate motifs for protein tyrosine kinases and discovery of anti-cancer agents.

### Local and systemic strategies for gene replacement for cancer

*JA Roth, SG Swisher, DD Lawrence, BL Kemp, CH Carrasco, AK El-Naggar, FV Fossela, BS Glisson, WK Hong, FR Khuri, JM Kurie, JC Nesbitt, K Pisters, JB Putnam, DS Schrump, DM Shin and GL Walsh*

Department of Thoracic and Cardiovascular Surgery, UTMD Anderson Cancer Center, Houston, TX 77030, USA

Advances in our understanding of the molecular genetics of lung cancer present an opportunity to develop prevention and treatment strategies based on the reversal of specific genetic lesions. Several experimental observations have suggested that correction of genetic abnormalities in the tumour cell could result in clinically significant tumour regression, and that corrective genes could be delivered by viral vectors. Viral gene transfer is more efficient in cancer cells than expected from studies in normal tissues. Viral vectors spread readily through tumours following single intratumoral injections. Transduced cells mediate bystander killing of non-transduced cells. Thus transduction of all tumour cells is not required as transduced cells can inhibit the growth of non-transduced cells. Human cancer cells have multiple genetic abnormalities. Correction of all these is not practical with current gene delivery systems. However, correction of critical single genetic lesions can cause regression of human tumours. Regional administration of viral vectors expressing wild-type tumour-suppressor gene *p53* prevents tumour growth for tumours with p53 mutations in orthotopic tumour models and mediates regression of large established tumours. These studies provided a rationale for clinical protocols to replace a defective *p53* gene with intratumour injection of recombinant retrovirus or adenovirus expressing normal *p53*. Patients (pts) who have failed conventional therapy with unresectable lung cancer obstructing a bronchus, extensive local regional tumour or isolated metastases that have a *p53* mutation undergo direct intratumour injections with the retroviral supernatant. Nine pts have been entered on this protocol. The presence of vector-derived *p53* DNA has been shown in post-treatment biopsies by polymerase chain reaction (PCR). In situ hybridization studies show nuclear localization of the vector. Post-treatment biopsies show increased apoptosis by TUNEL staining. Three pts have shown tumour regression. To date, no grade II or higher vector-related toxicity has been observed. We evaluated the safety and gene transfer efficacy of monthly intratumoral injections of a recombinant adenovirus containing wild-type p53 (Adp53) in 23 pts with advanced NSCLC who failed conventional treatments. Adp53 doses were escalated from $10^6$ plaque-forming units (pfu) in log increments to $10^{11}$ pfu. Pts were treated with or without cisplatin (CDDP) 80 mg $m^{-2}$ i.v. over 2 h given 3 days before Adp53 injection. Adp53 vector was injected monthly into the same primary or metastatic tumour under computerized tomography (CT) guidance. No significant vector-related toxicity was seen with up to 6-monthly injections. The severity of CDDP-associated toxicity (grade II or higher) was not increased with co-administration of Adp53. Clinical responses were evaluated with monthly abdominal and chest CT scans. Clinical responses with >50% regression of the injected tumour were observed and have been durable from 4 to 11+ months. To date, four major responses have been observed in the lung cancer trial. In addition, of the first 35 patients on whom complete data are available, 5 of 14 (36%) receiving $10^8$ Adp53 alone or higher doses had growth arrest of their tumour for ≥ 4 months, and 6 of 15 (40%) patients receiving Adp53 + CDDP had growth arrest of their tumour for ≥ 4 months despite three of the six progressing on CDDP or carboplatin previously. Post-treatment tumour biopsies demonstrated vector DNA by PCR, p53 transgene expression by reverse transcriptase-PCR and immunohistochemistry, increased necrosis on H and E sections and increased apoptosis by in situ TUNEL staining. Transgene expression occurred in post-treatment tumour biopsies in pts with circulating antibodies to adenovirus. There was no change in the inflammatory cells infiltrating the tumour, suggesting that non-specific inflammation was not responsible for the therapeutic effects. These results suggest that adenoviral mediated *p53* gene transfer can be successfully accomplished with minimal toxicity in pts with lung cancer. Adp53 clinical activity appears improved at higher doses of vector and when used in combination with CDDP.

### Tumour-selective targeting of prodrug activation

*N Lemoine*

ICRF Molecular Oncology Unit, Imperial College School of Medicine, Hammersmith Campus, London W12 0NN, UK

The principle of genetic prodrug activation therapy (GPAT) is to take advantage of the transcriptional differences between normal and tumour cells, or between the cells of one tissue and the rest of the individual. Expression of a gene that encodes a prodrug-activating enzyme is directed by a tumour- or tissue-specific gene promoter so that only these cells should become susceptible to the toxic effects of the prodrug when it is subsequently administered systemically. A variety of enzyme/prodrug systems are under development, all of which have advantages and disadvantages in particular applications. Most studies have been performed with the cytosine gene and the herpes simplex virus thymidine kinase gene.

Genes selectively expressed or overexpressed in cancer may be exploited as targets for therapy. The characterization of their transcriptional regulatory elements is crucial to the design of selectively expressed constructs. A variety of gene promoters might be exploited for GPAT in breast cancer, and already in our own laboratory encouraging results have been reported with the human ERBB2 promoter and MUC1 promoter.

Selective delivery may be simply achieved where there is an actively proliferating population of tumour cells in a quiescent background (brain tumours for instance) by using retroviruses that preferentially infect dividing cells. In other situations, selective delivery can be achieved by targeting cell surface receptors that mediate internalization. Many growth factor receptors are preferentially overexpressed in cancer, and some of these have been successfully exploited for ligand-mediated gene delivery.

*© Cancer Research Campaign 1998*

### Cancer vaccines: prospects and problems

*T Blankenstein*

Max-Delbrück-Center of Molecular Medicine, Robert-Rössle-Straβe 10, 13122 Berlin, Germany

Cytokines provided locally at the tumour site may initiate an effective antitumour immune response that leads to rejection of a tumour that would otherwise grow progressively. Experimentally, this can be tested by gene transfer into cultured tumour cells followed by the analysis of the tumorigenicity of such genetically engineered cells. This approach allows analysis of the function of a given cytokine in vivo and elucidation of the therapeutical value of genetically engineered tumour cells as vaccines. Our experience includes experiments with about ten cytokines, and the results can be summarized as follows: (1) some cytokines possess anti-tumour activity in this system, others do not; (2) a local and continuous cytokine supply seems to be essential for tumour rejection; (3) the tumour cell-derived cytokines act in a dose-dependent manner and in the absence of systemic toxicity; (4) the immunological effector mechanisms induced by different cytokines are partly cytokine specific, partly redundant (and usually involve T-cell-dependent and -independent mechanisms); (5) tumour rejection and mechanism thereof may be different with different tumour cell lines transfected with the same cytokine gene. Cytokine gene-modified tumour cells as vaccines are currently being tested in clinical trials. However, critical parameters, such as vaccine potency of cytokine gene-transfected tumour cells, optimal level of cytokine expression, reasons for varying vaccine effects in different tumour models, influence of irradiation of vaccine cells on their efficacy and attempts to improve vaccine efficacy (e.g. by coexpression of cytokines and T-cell co-stimulatory molecules, such as B7) have to be further addressed in experimental tumour models.

### Cancer therapy in 2020

*K Sikora*

WHO Cancer Programme, Lyon, France, and Department of Cancer Medicine, Imperial College School of Medicine, Hammersmith Hospital, London W12 0NN, UK

Over the next 25 years there will be major changes in the patterns of cancer incidence as the age distribution shifts in the population. Early detection and the ability to tailor treatments to the genetic characteristics of the tumour can be expected to improve cure rates. New diagnostic techniques involving molecular genetics will almost certainly allow the identification of high-risk groups for intensive screening and the application of preventive interventions. Early diagnosis will be enhanced by novel imaging technology, based on computerized three-dimensional reconstruction together with novel tumour markers.

The main modalities of treatment will improve. Surgery will result in increased organ preservation relying on minimally invasive techniques, computer-assisted virtual reality systems and robots. Eventually, surgery may even be replaced entirely with novel therapies. Radiotherapy will develop through multimedia imaging of tumour and normal tissues using computer optimization to obtain the best physical and biological therapeutic ratios. Designer fractionation based on individually predicted differential sensitivity to radiation will be possible through a greater understanding of the genes involved in DNA repair.

Novel systemic therapies are likely to have the greatest impact on cancer mortality. A new golden age of drug discovery is likely with the logical design of small molecules interfering with specific targets, such as signal transduction, transcription control, mitosis and apoptosis. The pace of acquisition of molecular knowledge on cancer is rapidly accelerating. The proteins encoded by both oncogenes and tumour-suppressor genes provide a promising hunting ground in which to design and test new anti-cancer drugs. Four to six genetic changes appear necessary to produce most human cancers. Over the next 5 years a much clearer understanding of the molecular pathogenesis of several specific malignancies is likely.

One of the greatest challenges will be caring for people with cancer, using the new technology in the face of declining organized religion and cohesive family structures. An increasing number of patients will neither die from their cancer nor be cured of it, but must learn to live with their disease, often being given complex therapies to control it for many years.

### The gene therapy industry – technology triumphs over common sense

*ME Gore*

The Royal Marsden Hospital, London SW3 6JJ, UK

All gene therapy strategies have to address three issues: the method of tumour cell kill, the choice of vector and the targeting of product to tumour cells. These three issues are not very different from those that face workers in the field of drug development. The problems that have to be overcome for successful cancer therapy are the same whether drugs or genes are used as a therapeutic. The ability to manipulate DNA sequences and insert them into cells in vitro with predictable effects has been very successful. Problems arise when attempts are made to translate these strategies into patients. The main areas of gene therapy are gene-directed enzyme prodrug therapy, corrective therapy and immunotherapy. There is a requirement in the first two strategies to have at least a very high proportion of tumour cells infected by the transgene without necessarily affecting normal cells. Most laboratory gene therapy programmes have concentrated on ways of killing tumour cells but this is not the major problem in cancer therapy. Tumour cell targeting is the key to successful cancer treatment, and practical ways of achieving this via gene therapy techniques are poorly developed. Many methods involve hybrid delivery systems using ligand binding technology that is often not exclusive to recombinant DNA technology. The strategy of achieving directed immunotherapy theoretically gets round the problem of specific tumour cell targeting, but the history of successful immunotherapy is not a glorious one. The most promising strategy for gene therapy involves tumour- and tissue-specific promotion, and it is this area of research that is most likely to lead to successful gene therapy approaches.

### Drug or gene therapy for ovarian cancer: which is the best hope for the future?

*SB Kaye*

CRC Department of Medical Oncology, University of Glasgow, Bearsden, Glasgow G61 1BD, UK

Ovarian cancer treatment is limited by the development of drug resistance, whose underlying mechanisms are not yet

© Cancer Research Campaign 1998

clarified. A key feature is failure of cells to undergo apoptosis after DNA damage, and this is linked to dysfunctional p53 in resistant cells. New agents, including taxoids and topoisomerase I inhibitors, can circumvent this resistance in vitro and do have clinical use, but they still have the classical problems of chemotherapy, i.e. a cellular target that is not selective for cancer cells.

The alternative approach, i.e. using tumour-specific promoters to provide selective gene therapy, has attracted considerable attention, but problems of delivery remain formidable. A novel form of 'genetic therapy' involves targeting of resistant tumour cells using the EIB genome-deleted attenuated human group C adenovirus Onyx-015. This can replicate selectively and then lyse cells with dysfunctional p53, and both in vitro and in vivo data confirm its activity in ovarian cancer models, including human tumour xenografts. Clinical trials using the intraperitoneal route are ongoing. Meanwhile experimental data indicate that optimal results may be obtained by a judicious combination of virus and drug therapy. Therefore, it is conceivable that, rather than choose between gene or drug therapy, the oncologist of the future will be fully employed using rational combinations of both forms of treatment.

### Selective cancer therapy with drugs – we've only just begun

*DR Newell*

Cancer Research Unit, University of Newcastle, Newcastle NE2 4HH, UK

Cancer is a genetic disease, and hence it is tempting to conclude that curing cancer will ultimately require gene therapy. However, cancer results from two types of gene defect, oncogene activation and tumour-suppressor gene inactivation, and gene therapy may not be relevant to both problems. Thus, while gene replacement to overcome tumour-suppressor gene malfunction is an obvious approach, with the exception of antisense strategies, it is not clear how gene therapy will correct problems resulting from proto-oncogene mutation, amplification or overexpression. In contrast, drug therapy can be used to both inhibit oncogene function and substitute for the actions of tumour-suppressor genes. For example, inhibitors of oncogenic tyrosine kinases are now in clinical trials, and small-molecule cyclin-dependent kinase inhibitors, to mimic the effects of natural inhibitors produced by p53 activation, are being developed. In addition to these conceptual attractions, small-molecule drugs can exploit a wealth of experience in medicinal chemistry, structural biology, preclinical and clinical pharmacology. Furthermore, small-molecule drug therapy avoids the problems of delivery inherent in gene-based approaches.

If there were currently no examples of curative therapy with small-molecule drugs, pessimism about the future of cancer chemotherapy would be warranted. However, experience with haematological malignancies, paediatric solid tumours and certain adult cancers has shown that curative cancer therapy with small molecules is feasible, albeit at levels and with toxicities that are not acceptable. Given that, for the first time, drugs targets are being exploited that are linked to the molecular pathology of cancer, there is genuine reason for optimism about future prospects for small-molecule drugs.

### Evidence for an immunomodulatory role for α-melanocyte-stimulating hormone (α-MSH) in cutaneous and ocular melanocytes

*SJ Hedley[1], M Wagner[1], DJ Gawkrodger[2], AP Weetman[1], IG Rennie[3] and S MacNeil[1]*

University Departments of [1]Medicine, [2]Dermatology, and [3]Ophthalmology and Orthoptics, Sheffield S5 7AU, UK

α-MSH, a defined pigmentary hormone in many species, has a broad spectrum of biological actions, including the reduction of experimentally induced fever in animals. We speculated that it may assist local melanocytes to resist UV-induced damage and local inflammation. Such an action could prevent melanocytes becoming a premature target for the immune system. In melanoma, this mechanism could help metastatic cells escape the attentions of the immune system.

We investigated the effects of α-MSH on TNF-α stimulated ICAM-1 expression, as ICAM-1 is one of several ligands required for T-cell adherence and activation. Cutaneous and ocular melanocytes were expanded in culture using a mitogen-rich MCDB153 medium. Five to ten days before ICAM-1 analysis, mitogens were removed from the medium, as this was necessary to demonstrate any response to α-MSH. Unstimulated melanocytes had constitutively low ICAM-1 expression, which was up-regulated by the addition of TNF-α ($100\ \mathrm{U\ ml^{-1}}$) for 24 h but unaffected by the addition of α-MSH alone. However, co-incubation of cells with α-MSH and TNF-α significantly reduced TNF-α-stimulated ICAM-1 response by approximately 50% ($10^{-11}$ M α-MSH reduced the response to TNF-α by 35 ± 11%, $n = 5$); $10^{-8}$ M α-MSH by 57 ± 8%, $n = 8$). Maximal responses to α-MSH were usually seen between $10^{-10}$ and $10^{-8}$ M α-MSH for cutaneous melanocytes. In ocular melanocytes, preliminary data showed that $10^{-10}$ M α-MSH reduced the ICAM response to TNF-α by 55% in one experiment but only by 5% in a second. IBMX mimicked the α-MSH response by significantly reducing TNF-α-stimulated ICAM-1 expression in both cutaneous and ocular melanocytes. This implicates the involvement of cyclic AMP in the mechanism.

Present data strongly suggest that α-MSH has the potential to oppose the actions of inflammatory cytokines in melanocytes. We suggest that this is probably part of the normal spectrum of the biological actions of α-MSH in skin and possibly the eye. Melanoma cells are known to produce α-MSH (and related POMC peptides). If a similar response to α-MSH is retained in metastatic melanoma cells, this could oppose the action of cytokines released from the immune cells.

### Coexpression of bcl 2 with herpes simplex virus thymidine kinase in tumour cells increases non-apoptotic cell death on ganciclovir treatment

AA Melcher[1], *MD Jacobson[2], and RG Vile[1]*

[1]Imperial Cancer Research Fund Molecular Oncology Unit, RPMS, Hammersmith Hospital, Du Cane Road, London, W12 0NN; [2]MRC Laboratory for Molecular Cell Biology, UCL, Gower Street, London, UK

We have previously demonstrated that killing tumour cells in vivo with the HSVtk/ganciclovir (GCV) system generates anti-tumour

 © Cancer Research Campaign 1998

immunity. However, the strength of this immunity differs between cell lines; for example, B16tk+ tumours uniformly generate increased protection against parental cell rechallenge (40–70% protection) compared with CMT93tk+ tumours (10–20% protection). While investigating the mechanisms involved in these effects, we observed that GCV-mediated cell killing of the B16 melanoma cell line occurs predominantly via necrosis (<1% of apoptotic cells per field after 48 h of GCV treatment in vivo). In contrast, killing of the colorectal CMT93 cell line occurred with increased levels of apoptosis (20% of cells per field). These findings were confirmed by studying the mechanisms of cell killing in vitro using DNA ladders, EM and propidium iodide staining at several time points after GCV treatment. Therefore, we proposed that necrotic cell death in vivo may provide a potent immunostimulatory signal that serves as a 'danger' signal to allow the breaking of tolerance to tumour antigens. Hence, the degree to which HSVtk killings leads to necrotic or apoptotic cell death may influence the subsequent strength of the anti-tumour immunity.

To test this hypothesis, we transfected the CMT93tk line with a retrovirus encoding *bcl* 2 in an attempt to block apoptotic cell death during HSVtk/GCV killing. A population of CMT93tk cells, in which a proportion of cells express *bcl*-2 by FACS, are still sensitive to GCV in vitro but show greatly reduced amounts of apoptotic cell death as judged by DNA ladders and propidium iodide staining. Data will be presented on the use of these cells in in vivo experiments to determine whether the immunogenicity generated by GCV killing of the bcl-2+ cells is greater than that of the parental CMT93tk line. The implications of these findings for the development of the 'danger' hypothesis in cancer immunogene therapy will be discussed.

### Concordance between epithelial tumour cell metabolic activity and susceptibility to chemo- and radiosensitivity

*AME Nouri[1], MV Russell[1], H Cannell[2], AMI Paris[3], and RTD Oliver[1]*

Departments of [1]Medical Oncology, [2]Maxillofacial Surgery and [3]Urology, London Hospital, London E1 1BB, UK

Colorimetric and immunocytochemical staining techniques were used to assess the influence of hyperthermia and, in parallel, the relevance of epidermal growth factor receptor (EGFR) expression to radio- and chemosensitivity of four human epithelial tumour cell lines of different origins.

The results showed that: (1) all cell types showed inhibition of metabolic activity as measured by optical density (OD) in response to cisplatin or gamma radiation in a dose-related manner; (2) increase in culture temperature or stimulation of cells expressing high intensity of EGFR with EGF resulted in an increase of cellular metabolic activity by as much as 60% (the stimulatory activity of EGF was significantly decreased when temperature was increased from 37°C to 40°C); (3) addition of EGF to cells increased their susceptibility to cisplatin by up to 40%; and (4) increasing temperature from 37°C to 40°C resulted in an increased sensitivity of cells to cisplatin and radiation by up to 35% and 25% respectively.

These results indicate the relevance of status of cell aggressiveness as indicated by their high levels of metabolic activity and their responsiveness to radio- and chemotherapy. The induction of hyperthermia should increase the efficacy of cancer treatment modalities. This will make it possible to treat patients with lower concentration of drugs to achieve the same level of response. In addition, the finding that the higher-EGFR-expressing tumour cell lines responded more efficiently to treatment when exposed to EGF might provide a lead for the better selection of patients for chemotherapy.

### DNA Interaction with polymeric vesicles – consequences for the transfection efficiency (in vitro)

*A Schätzlein[1], M Brown[2], S. Siddique[2], IF Uchegbu[2], and L Tetley[3]*

[1]CRC Department of Medical Oncology, Beatson Laboratories, University of Glasgow, Glasgow G61 1BD; [2]Department of Pharmaceutical Sciences, University of Strathclyde, Glasgow G1 1XW; [3]EM Centre Biological Laboratory, ILBS, University of Glasgow; Glasgow, G12 8QQ, UK

Although various promising strategies for the genetic therapy of cancer have been proposed recently, the specific and efficient delivery of the genetic material into (cancer) cells still remains a major hurdle. Viral vectors, e.g. adenoviruses, have been shown to be quite efficient and even specific vectors, but virus-related toxicity and immune response impose severe restrictions on their (repeated) use (Blaese et al, 1995, *Cancer Gene Therapy* **2**: 291). However, the efficiency of non-viral vectors, most of them based on cationic amphiphiles (Gao and Huang, 1995, *Gene Therapy* **2**: 710) and polymers (Stankovics et al, 1994, *Human Gene Therapy* **5**: 1095), remains unsatisfactory for many in vivo applications. We advocate the use of modular non-viral gene delivery systems that consist of a combination of tools that fulfil dedicated functions. Vesicles are one option as basis for such modular systems.

Novel polymeric vesicles have been prepared from prepolymerized amphiphiles, such as glycol chitosan (degree of polymerization ~ 800). Their biocompatibility was studied by a haemolysis assay and MTT assay (Plumb et al, 1989, *Cancer Res* **49**: 4435) with three human cell lines (A2780, A549 and A431). The interaction with DNA was studied in combination with cationic amphiphiles (e.g. DOTAP, DODAB) and alone. The morphology of the complexes was characterized by freeze fracture EM. The degree of DNA condensation was determined by ethidium bromide exclusion. Zeta potential measurements and gel retardation assays were used to study their net charge and electrophoretic mobility (zeta potential, gel retardation). The transfection efficiency of the DNA–carrier complexes was studied with reporter gene constructs (pCMV-Sport-βGal, pEGFP-C1) in A549, A2780 and A431 cells in vitro.

The self-assembly of preformed polymers into vesicles was assumed to depend on the decoupling of the polymer backbone from the membrane-forming amphiphile. However, we have found that carbohydrates, such as glycol chitosan and other biopolymers, once modified by the attachment of fatty acid pendant groups (GP41), may self-assemble and form unilamellar polymeric vesicles in the presence of cholesterol. Such systems may be produced from the hydrophobic modification of a wide variety of carbohydrates, such as starch, dextran and other oligosaccharides. Such vesicles may be useful drug carriers as they have an inherently greater membrane stability then conventional vesicles. They were stable and retained

© Cancer Research Campaign 1998

approx. 60% of their drug content (water-soluble glycopeptide, $M_r$ 1500) over a period of more than 5 weeks. GP41 vesicles did not induce significant haemolysis at erythrocyte/GP41 ratios of 300–30:1 w/w (DOTAP 80–100% haemolysis) and were biocompatible with human cells (A2780, A549 and A431), with no toxicity evident at concentrations of GP41 ≤ 150 μg $ml^{-1}$. DNA condensation by GP41/cholesterol polymeric vesicles was only moderate (20–40% at amphiphile/DNA molar ratios of 0.25–5:1) compared with a condensation of approx. 90% achieved by DOTAP (ratio 2.5–5:1). When combined with the cationic amphiphile DODAB, however, DNA/DODAB-GP41/cholesterol complexes were able to achieve a condensation of 90%. Cationic amphiphiles were significantly less toxic when combined with polymeric vesicles. Polymeric vesicles were not able to transfect mammalian cells in vitro when used alone but could be tailored to deliver DNA to cells in combination with cationic amphiphiles. The presence of cationic amphiphiles is probably necessary to improve DNA condensation but may also influence the endosomal escape of the DNA carrier complex later on. The combination of polymeric vesicles with other compounds into a 'shuttle' vector may help to improve non-viral gene delivery in vivo.

### Influence of tumour cell proliferation on expression of cellular phosphatases

*AME Nouri, AANM Dabare and RTD Oliver*

Medical Oncology, London Hospital, London E1 1BB, UK

Cytometric, immunocytochemical, biochemical and colorimetric (MTT) assays were used to assess the influence of cell proliferation on the expression of cellular alkaline phosphatases (ALP) in established human tumour cell lines of epithelial origin. These enzymes are increasingly being recognized as important markers for monitoring tumour cell behaviour in some human malignancies.

The results showed that:

(1) The degree of ALP differs widely in various tumour cell lines. Based on biochemical analysis, the profile of ALP fell mainly into two categories: (a) low expressing (MW 72 kDa, most probably placental type ALP), such as Hep2 and KB (head and neck); and (b) those that expressed both low- and high-molecular-weight (MW 95 kDa) bands, i.e. mainly tumours of testis, including Tera II and Ep2012.
(2) In all the cases, treatment of tumour cell lysates with heat before biochemical analysis showed the disappearance of the higher MW and sharpening of the lower band.
(3) Exposure of tumour cells to epidermal growth factor (EGF) led to an increased cellular proliferation as measured by MTT assay. In parallel, there was a concomitant decrease in ALP bands as assessed by biochemical method.
(4) Cytometric analysis of cells comparing expressed ALP as detected by monoclonal antibody specific for placental-type ALP showed that there was as much as 77% decrease in the expression when cells were exposed to EGF in culture. In addition, this was only observed in cells expressing high-intensity EGFR.

These findings demonstrate the differential expression of ALP in tumour cell lines of different origins. The results also indicate that, as the status of cell proliferation increased, there was a concomitant decrease in the levels of ALP expression. These issues raise the question as to whether the differential expression of EGFR and ALP in tumour biopsies may provide an indication of tumour aggressiveness, a study that is currently under investigation.

### The cell death–cell cycle interface: biochemical mechanism and physiological role

*HJM Brady[1], O Williams[1], A Berns[2], D Kioussis[1] and G Gil-Gómez[2]*

[1]Division of Molecular Immunology, NIMR, Mill Hill, London NW7 1AA; [2]Division of Molecular Genetics, Netherlands Cancer Institute, Plesmanlaan 121, 1066 CX Amsterdam, The Netherlands

There are no decisions more fundamental to the cell than whether to divide or to die? When cell division becomes deregulated, tumour formation and cancer can be the result. Furthermore, if cells lose the ability to die by apoptosis in response to normal physiological cues, this may also contribute to tumour formation. Therefore, the possibility that the pathways of cell cycle and apoptosis may have an overlapping biochemical mechanism is of great fundamental interest, as well as having potentially important therapeutic implications for disease, including cancer.

We have approached the cell cycle–apoptosis interface from our work on the Bax and Bcl-2 molecules, which regulate cell death. We have shown that, although p53 regulates expression of the bax gene, overexpression of Bax in p53–/– thymocytes is insufficient to restore the apoptosis pathway lost in the absence of wild-type p53 (Brady et al, 1996*a*, *EMBO* **15**: 1221). We further showed that, in our bax transgenic mice, T-cell development is severely disrupted, which implies a role for Bax/Bcl-2 in the production of a fully functional immune system. Using T cells from bax and bcl-2 transgenic mice, we also showed that Bax accelerates while Bcl-2 slows down entry into the cell cycle (Brady et al, 1996*b*, *EMBO* **15**: 6991). We have now shown that resting cells triggered to die down-regulate $p27^{Kip1}$, an event normally associated with cell cycle entry. We also show that Bax/Bcl-2 can directly influence the extent of $p27^{Kip1}$ degradation and hence the activity of cyclin-dependent kinase (Cdk) 2. Specific pharmacological inhibitors of Cdk2 activity can completely block various forms of apoptosis but not that induced via the CD95 (Fas) receptor. We show that the activity of Cdk2 in apoptosis is not associated with its canonical cyclins (A and E) as during the cell cycle. We also describe experiments that directly prove the influence of Cdk2 activity alone as an effector of apoptosis. Using fetal thymic organ culture from transgenic mice in the presence of various Cdk2-specific inhibitors, we show the importance of Cdk2 activity in various physiologically important cell death pathways, such as antigen-driven negative selection. Bcl-2 acts as an oncogene, and recently Bax has been shown to be a tumour-suppressor gene. Bax/Bcl-2 ratios have been shown to be good prognostic indicators of various forms of cancer. We discuss our cell cycle–cell death data in the light of these facts and their therapeutic implications for cancer treatment.

### The *tpr* gene is overexpressed and inducible in ovarian cancer cells exposed to carboplatin

*P Gilmore, MT Cairns, P White, S Church and PG Johnston*

Department of Oncology, Belfast City Hospital, BT9 7AB, UK

The development of platinum resistance is a common occurrence in ovarian cancer. We have used the differential display (DD) technique (Liang and Pardee, 1992, *Science* **257**: 967) on paired platinum-sensitive and -resistant ovarian epithelial cell lines (PEA1/PEA2 and PEO1/PEO4) to identify genes with a putative

© Cancer Research Campaign 1998

involvement in the development of platinum resistance. Growth inhibition assays showed that PEA2 cells ($IC_{50}$ $1.5 \times 10^{-6}$ M ± 0.17) were ten times more resistant to carboplatin (CBDCA) than PEA1 ($IC_{50}$ $1.97 \times 10^{-7}$ M ± 0.098, while PEO4 cells ($IC_{50}$ $6.3 \times 10^{-6}$ M ± 0.86) were 40 times more resistant to CBDCA than PEO1 ($IC_{50}$ $1.99 \times 10^{-6}$ M ± 0.22). Differential display patterns were obtained from PEA1/PEA2 and PEO1/PEO4 cells grown under normal culture conditions and after a time-course (1, 6 and 24 h) of CBDCA exposure. Northern blotting confirmed the differential expression of a candidate gene identified by DD, which hybridized to a specific mRNA of 3.5 kB in size. Sequence analysis revealed homology (92%) to the *tpr* (*t*ranslocated *p*romoter *r*egion) gene sequence (Mitchell and Cooper, 1992, *Oncogene* **7**: 383). Expression of the *tpr-homologous* ('*tpr-hom*') gene was found to be increased more than threefold in untreated platinum-resistant cell lines (PEA2 and PEO4) compared with sensitive cell lines (PEA1 and PEO1) and was induced over a time-course of acute exposure to $IC_{50}$ concentrations of CBDCA in the same cell types (by threefold and 12-fold in PEA2 after 6 h and 24 h, respectively, and by sixfold and 38-fold in PEO4), while remaining unchanged from control levels in the sensitive cells. These data suggest that *tpr* may play an important role in the development of platinum resistance, which will be further investigated via transfection of a full-length *tpr* cDNA (clone obtained courtesy of Dr L Gerace) into the sensitive (PEA1 and PEO1) types to determine its effect on sensitivity/resistance phenotype.

### The development of a quantitative RT-PCR for thymidylate synthase from formalin-fixed and paraffin-embedded tissue specimens

*S Church, P Gilmore, P White and PG Johnston*

Department of Oncology, Belfast City Hospital, BT9 7AB, UK

The expression level of the thymidylate synthase (TS) enzyme has been established as a predictor of response to 5-fluorouracil (5-FU)-based therapy. Patients whose tumours express low levels of TS respond significantly better than those with high TS levels. Quantitative reverse transcriptase–polymerase chain reaction (RT-PCR) and immunological quantitation have been adopted as techniques to measure TS levels in tumour samples. In an attempt to improve the applicability of these techniques, we have developed a novel RT-PCR technique for TS quantitation using formalin-fixed and paraffin-embedded (FFPE) tissue.

Total RNA was isolated from FFPE sections ranging in size from 16 to 64 microns. Spectrophotometrically detectable RNA was obtained using 30-micron amounts of starting material. The amounts of RNA obtained ranged from a minimum of 0.5 μg to a maximum yield of 8 μg. The β-actin gene was chosen as the internal PCR standard, using primer sequences designed to generate a 230-bp amplicon; the TS-specific primers yield a 250-bp amplicon. Both the β-actin and TS target sequences were readily amplifiable from cDNAs generated from archival RNA samples. That these products were derived from cDNA rather than genomic DNA contamination was confimed by the use of primers for the thymidine kinase (TK) gene, which span an intron and yield a 96-bp amplicon from cDNA samples and a 206-bp amplicon from genomic DNA. Optimization experiments determined that a PCR cycle number of between 22 and 25 rendered both the β-actin and TS products in the log phase of amplification. The procedure for performing quantitative RT-PCR on RNAs isolated from archival tissue has been optimized and is suitable for use in further investigations that will compare levels of TS determined by RT-PCR with immunohistochemical analysis from the same pathological tissue sample.

### Application of the amplification refractory mutation system (ARMS™) with real-time fluorescence detection to assess K-*ras* mutations in bronchoalveolar lavage (BL) specimens for early lung cancer detection

*FM Scott[1,2], SJ Clayton[3], K Callaghan[3], W Prime[1,2], M Walshaw[4], L Turnbull[5], G Ellison[3], JC Fox[3] and JK Field[1,2]*

[1]Roy Castle International Centre for Lung Cancer Research, 200 London Road, Liverpool L39TA; [2]Molecular Genetics and Oncology Group, Clinical Dental Sciences, University of Liverpool, L69 3BX; [3]Zeneca Diagnostics, Gadbrook Park, Northwich, Cheshire CW9 7RA; [4]Cardiothoracic Centre, Broadgreen Hospital, Thomas Drive, Liverpool L14 3LB; [5]Department of Pathology, University of Liverpool, L69 3BX, UK

Mutations of the *ras* family of oncogenes are the most frequently observed genetic lesions in a number of human malignancies. About 80–90% of activating mutations of the K-*ras* gene occur at codon 12 or 13 in human non-small-cell lung cancer. K-*ras* mutations therefore provide a screening target for early detection of lung cancer. Seven ARMS tests have been developed for activating mutations at codons 12 and 13 of K-*ras*. Application of ARMS to lung tumour DNA has previously identified 9% of non-small-cell lung cancers as possessing K-*ras* mutations (Neville et al, 1995, *Int J Oncol* **7**: 511). One hundred and eleven BL DNA samples from patients with lung-related problems were analysed for K-*ras* mutations; 75 of the samples were initially analysed by gel electrophoresis of ARMS-generated PCR products and three were identified with K-*ras* mutations (codon 12 Gly→Asp). Although these specimens were negative for cancer by cytology, one was from a patient with a diagnosis of secondary adenocarcinoma of the lung and two were from patients with non-cancer diagnoses. The other 36 BL DNAs were analysed using homogenous fluorescence-based detection of ARMS-generated PCR products in a real-time format. Four mutations (three Gly→Cys, one Gly→Val at codon 12) were detected in these samples. Two were in BL specimens from patients diagnosed with lung adenocarcinoma, and these specimens also contained adenocarcinoma cells by cytology. The other two were from patients with a diagnosis of lung carcinoma, but with no cancer cells detected by cytology. The results of the ARMS analyses will be presented and the potential of K-*ras* testing for lung cancer screening will be discussed.

### Microsatellite instability and loss of heterozygosity in bronchial lavage specimens

*T Liloglou[1,2], G Xinarianos[1,2], W Prime[1,2], M Walshaw[3], L Turnbull[4] and JK Field[1,2]*

[1]Roy Castle International Centre for Lung Cancer Research, Liverpool L3 9TA; [2]Molecular Genetics and Oncology Group, Department of Clinical Dental Sciences, The University of Liverpool, Liverpool L69 3BX; [3]The Cardiothoracic Centre, Broadgreen Hospital, Liverpool L14 3PE; [4]Department of Pathology, The University of Liverpool, Liverpool L69 3BX, UK

© Cancer Research Campaign 1998

Genomic instability is now considered to be a hallmark of cancer. Analysis of microsatellite markers in tumour tissue compared with its normal counterpart has become the most widely used method to determine genomic instability in the form of microsatellite alterations (MA) or loss of heterozygosity (LOH). Genomic instability has been found in 42–49% of non-small-cell lung cancers (Miozzo et al, 1996, *Cancer Res* **56**: 2285; Neville et al, 1996, *Int J Oncol* **9**: 533). We studied 90 bronchial lavage specimens from individuals with suspected lung cancer using 12 microsatellite markers. Genomic instability was found in 15 of 43 (35%) patients with lung cancer, however it was also found in 11 of 47 (23%) patients with no cytological or radiological evidence of bronchial neoplasia. We have also demonstrated the same genomic instability in a number of bronchial lavage specimens and in the corresponding tumours. These results demonstrate the potential of MA in bronchial lavage specimens as an early detection marker.

This research is supported by the Roy Castle Cause for Hope Foundation.

### Telomerase activity in lung cancer and human bronchoalveolar lavage fluid

*G Xinarianos[1,2], FM Scott[1,2], W Prime[1,2], M Walshaw[3], L Turnbull[4] and JK Field[1,2]*

[1]Roy Castle International Centre for Lung Cancer Research, 200 London Road, Liverpool L39TA; [2]Molecular Genetics and Oncology Group, Clinical Dental Sciences, University of Liverpool, L69 3BX; [3]Cardiothoracic Centre, Broadgreen Hospital, Thomas Drive, Liverpool L14 3LB; [4]Department of Pathology, Duncan Bldg., University of Liverpool, L69 3BX, UK

The maintenance of telomeres, or chromosome ends, is essential to the progression of cells through a normal, mortal life span. In tumour tissues and immortalized cells, telomeres are maintained by telomerase, a ribonucleoprotein polymerase, at lengths that are generally shorter than normal tissue. Telomerase is expressed in most cancers and absent in most normal tissues. This includes about 80% of primary lung tumours, about 93% of breast cancers, 90% of head and neck squamous cell carcinomas and 88% of cervical cancer specimens. We have measured telomerase activity in lung cancer cell lines and lung tumours using the telomerase repeat amplification protocol (TRAP) (Kim et al, 1994, *Science* **266**: 2011). Four of four lung cancer cell lines and 12 of 15 lung tumours examined contained telomerase activity, which was not detectable in matching normal tissue. In order to investigate telomerase as a potential early detection marker for lung cancer, the enzyme will need to be measured in bronchoalveolar lavage (BL) or sputum specimens. Three of 19 (16%) BL specimens from patients undergoing bronchoscopy for diagnosis of lung cancer and for other reasons had detectable telomerase activity by the TRAP, applied in a radioactive or non-radioactive format. The possibility of inclusion of telomerase in a panel of lung cancer early detection markers will be discussed.

This research is supported by the Roy Castle Cause for Hope Foundation.

### A 'new' glioma-associated cell surface glycoprotein

*K Mulligan[1], D McCormick[2], E Dermott[3], B Wisdom[4], B Radotra[1] and M Mirakhur[1]*

[1]Neuropathology, [2]Oncology, [3]Microbiology and [4]Biochemistry, The Queen's University of Belfast, Belfast BT9 7AB, UK

While malignant gliomas rarely metastasize, they characteristically exhibit diffuse and extensive invasion into normal brain parenchyma. This invasive capacity is primarily responsible for the failure of current therapies to prevent tumour recurrence. Glioma invasion is a complex process involving partial degradation of the extracellular matrix (ECM) and cell–cell and cell–ECM interactions mediated by cell surface adhesion molecules.

As part of an investigation of glioma adhesion molecules, monoclonal antibodies were generated to proteins expressed by an anaplastic astrocytoma cell line. One of the hybridomas produced secretes an antibody (MQ1) that recognizes two previously undescribed mammalian glycoproteins of molecular weight 50 kDa and 200 kDa. N-terminal sequencing revealed that both proteins have the same N-terminal amino acid sequence and that they share 74% homology with a micro-organism adhesion protein.

Immunoprecipitation demonstrated that the 200-kDa protein was expressed on the cell surface of astrocytoma cells in vitro.

Immunocytochemistry showed expression of the MQ1 surface protein on a range of fetal astrocytes and astrocytoma cell lines in vitro, and flow cytometry indicated that the level of expression correlated with astrocytoma malignancy and inversely correlated with fetal astrocyte maturity.

Immunohistochemistry on histologically normal and neoplastic brain tissue demonstrated that in the central nervous system (CNS) MQ1 protein expression is restricted to cells of astrocytic lineage, and that reactive astrocytes and astrocytomas are strongly immunoreactive for the MQ1 proteins.

In summary, the 200-kDa MQ1 protein is a previously undescribed glioma-associated cell-surface glycoprotein, with properties suggesting that it may be an adhesion molecule with a role in fetal development and neoplasia in the CNS.

### Antiproliferative effects of bioactive peptides secreted by frog skin towards human breast cancer cells in vitro

*HW van den Berg[1], C Shaw[2], M Lynch[1] and P Johnston[1]*

Departments of [1]Oncology and [2]Medicine, The Queen's University of Belfast

Drugs derived from natural products play an important role in the treatment of disease, including cancer. However, until recently, large-scale, systematic and rational drug discovery has not been undertaken. We have initiated studies of the antiproliferative properties of natural peptides from a number of species, including those contained in dermal venom components of frogs.

The antiproliferative effects of three purified and sequenced peptides (frog mellitin, PEC and PLR) have been investigated using a panel of human breast cancer cell lines (ZR-75-1, ZR-PR-LT, ZR-75-9al, MCF-7 and LY2), which differ in their anti-oestrogen sensitivity, steroid hormone receptor profile and in their expression of epidermal growth factor receptors (EGFR) and insulin-like growth factor-I receptors (IGFR). Inhibition of cell proliferation was assessed over a 9-day period of growth in medium supplemented with 5% fetal calf serum in 96-well plates using the MTT colorimetic assay.

Frog mellitin failed to significantly influence the proliferation of any of the cell lines investigated. PEC at concentrations ranging from $10^{-10}$ to $10^{-8}$ M stimulated the proliferation of oestrogen-dependent ZR-75-1 and tamoxifen-resistant LY2 cells, while

© Cancer Research Campaign 1998

failing to consistently influence the growth of the other cell lines in the study. In contrast, PLR in the concentration range $10^{-12}$–$10^{-8}$ M inhibited the proliferation of all the cell lines studied. A reduction in cell number was observed 3, 6 and 9 days after treatment with drug replenishment on days 3 and 6. In ZR-75-1, MCF-7 and the tamoxifen-resistant ZR-75-9a1 lines, this inhibition was modest (20–40%), non-dose dependent and cells showed signs of recovery at day 9. However, inhibition of proliferation of ZR-PR-LT and LY2 cells was clearly dose dependent, with $IC_{50}$ values at day 9 of approximately $10^{-8}$ M. Interestingly, these cell lines have reduced numbers of EGFR in comparison to their respective parent lines. This may reflect receptor occupancy, raising the possibility that PLR may act in part by interfering with EGF/TGF alpha action at the EGF receptor.

We conclude that PLR shows promise as an antiproliferative peptide, and that the effects observed are unlikely to be non-specific as response was variable among the cell lines studied. Further studies will be designed to determine the antiproliferative mechanism of PLR and assess its ability to bind to specific cell membrane-associated receptors.

### The expression of desmoglein-2 in primary breast cancer and its relation to a series of prognostic markers

*EL Davies[1], JMW Gee[1], RA Cochrane[1], RI Nicholson[2], AK Sharma[1], WG Jiang[1] and RE Mansel[1]*

[1]Department of Surgery and [2]Tenovus Cancer Centre, University Hospital of Wales, Cardiff, UK

The presence of local invasion and distant metastasis is of prime importance to patients presenting with primary breast cancer. Several adhesion molecules (i.e cadherins) have been shown to act as metastatic suppressor molecules. Desmoglein 2 (Dsg2) is a novel glycoprotein forming a subfamily of the cadherin group of adhesion molecules. Its role as a potential metastatic suppressor molecule has been demonstrated in transitional and squamous cell carcinomas and in our recent breast cancer in vitro data. However, its expression and role in breast cancer in vivo has yet to be established.

The aims of this study were to demonstrate and establish the level of immunohistochemical (IHC) expression of Dsg2 in breast cancer specimens ($n$ = 68) as well as in ductal carcinoma in situ (DCIS) ($n$ = 3), tumour-associated normal (TAN) ($n$ = 11) and normal breast tissue ($n$ = 9). Secondly, we compared the IHC level of Dsg2 expression and a series of prognostic markers [oestrogen (ER) and progesterone (PR) receptors, Ki-67, epidermal growth factor receptor (EGFR), grade and size of tumour, lymph node involvement and menopausal status].

The results revealed that the majority of cancers (98.5%) demonstrated membranous staining for Dsg2. However, the Dsg2 expression was significantly reduced in breast cancer compared with normal and TAN ($P < 0.0001$) but not with DCIS ($P = 0.067$). A significant inverse relationship between tumour size and Dsg2 expression was demonstrated ($P = 0.012$), but no correlation could be shown between Dsg2 level and the other prognostic markers.

These results suggest that Dsg2 may have a potential in local breast cancer progression, but have not provided evidence for a metastatic suppressor role for Dsg2.

### Effects of in vivo interleukin-12 (IL-12) administration in renal cell carcinoma patients

*D Stark, RE Banks, RW Carter, MA Forbes, S Hallman, J Stanley, PJ Selby and PM Patel*

ICRF Cancer Medicine Research Unit, St James's University Hospital, Leeds LS9 7TF and UK

Six patients (three male and three female) with inoperable renal cell cancer received recombinant human IL-12 as part of a multi-centre phase II European trial. After dose escalation, 0.1, 0.5, 1.25 μg/kg$^{-1}$ s.c. weekly over the first 3 weeks, these were up to three further cycles of treatment, each consisting of three weekly injections (1.25 μg kg$^{-1}$ s.c.) and one rest week. Four patients had no significant clinical problems. One patient developed pulmonary oedema during a blood transfusion. One patient developed the nephrotic syndrome. The relationship of these events to IL-12 is unknown and under investigation. One patient had a WHO grade 3 or higher rise in their liver biochemistry, and two had a grade I elevation. Three patients had WHO grade I chills and one of the six had grade I oral mucositis. Two patients stopped treatment because of adverse events, two because of disease progression, and two had stable disease at the end of treatment.

Serum level of IL-12 ranged from 40 to 450 pg ml$^{-1}$, by ELISA, 48 h after dose one, and returned to under 150 pg ml$^{-1}$ by 24 h. PBMC subpopulations and lymphocyte activation were measured by flow cytometry. Increased NK and LAK activity were demonstrated after IL-12. $T_H1/T_H2$ cytokine balance was assessed by three-colour cytometric analysis of intracellular cytokines. IFN-γ was induced, and serum levels appeared to alter in a dose-dependent manner.

### PDEPT: polymer-directed enzyme prodrug therapy

*R Satchi and R Duncan*

Centre for Polymer Therapeutics, The School of Pharmacy, University of London, London WC1N 1AX, UK

Improvements in cancer chemotherapy, particularly for the treatment of common solid tumours, have been difficult to realize. PDEPT is a novel approach using polymeric prodrugs and polymer-bound enzymes to improve cancer therapy. The concepts of antibody-directed enzyme prodrug therapy (ADEPT) and gene-directed enzyme prodrug therapy (VDEPT) are already well established and have demonstrated improved therapeutic activity in animal models and pilot human studies. However, both approaches have a number of inherent limitations, such as immunogenicity, difficulty in optimization of the dosing schedule because of the plasma half-life of antibody enzymes, the need to use a clearing antibody and the inherent dangers associated with a viral vector.

PDEPT uses a polymer–enzyme conjugate that accumulates selectively in solid tumour tissue by the enhanced permeability and retention (EPR) effect to selectively activate a polymeric prodrug that can likewise accumulate preferentially in the tumour. The polymeric prodrug can be designed to contain a potent cytotoxic agent covalently bound to polymer via a linker designed for cleavage by the enzyme present in the polymer–enzyme conjugate. PDEPT has the potential to overcome the problems

© Cancer Research Campaign 1998

associated with ADEPT and VDEPT. *N*-(2-hydroxypropyl)methacrylamide (HPMA) co-polymer enzyme conjugates will have little or no immunogenicity (Flanagan et al, 1990, *J Bioact Compat Polymers* **5**: 151), and HPMA co-polymer–doxorubicin conjugates have already shown promise in an early clinical trial (Vassey et al, 1996, *Ann Oncol* **7**: 97). In this study, we have prepared and characterized HPMA polymer conjugates containing β-lactamase and cathepsin-B as model enzymes, with a yield of 28%. Activity of free and conjugated enzymes was assayed using low-molecular-weight prodrugs (benzylpenicillin for β-lactamase and Bz-Phe-Val-Arg-p-nitroaniline for cathepsin-B) as well as on high-molecular prodrugs such as HPMA-Gly-Phe-Leu-Gly-doxorubicin (PK1) for cathepsin-B. Both retained enzyme activity after polymer conjugation giving $V_{max}$ 0.2 nM $s^{-1}$ $unit^{-1}$ and $K_m$ 0.1. Radiolabelled β-lactamase and HPMA-Gly-Gly-β-lactamase were administered i.v. to C57 mice bearing s.c. B16F10 melanoma for comparison of their pharmacokinetics. Polymer–enzyme conjugate showed a retention time of 3.6-fold in the blood with respect to the free enzyme and higher site-specific passive capture through the EPR effect giving twofold increase in accumulation of the conjugate compared with free enzyme in the tumour.

### Preferential replication of an E1B-attenuated adenovirus in drug-resistant ovarian tumour lines with defective mismatch repair and loss of p53 function

*YT Kim, I Ganley, M Illand, S Kaye and R Brown*

CRC Department of Medical Oncology, CRC Beatson Laboratories, Glasgow G61 1BD, UK

Compared with wild-type Ad2 virus, an adenovirus lacking E1B 55-kDa gene expression (ONYX-015) has significantly reduced replication in the wild-type p53-expressing human ovarian adenocarcinoma A2780 cell line. We have measured virus replication using plaque-forming assays and expression of viral hexon proteins in infected cells. Introduction of a mutant p53 (codon 143, Val to Ala) into these A2780 cells causes loss of p53 function and increased replication of ONYX-015. Defective mismatch repair and loss of p53 function is associated with resistance to the anticancer drug cisplatin in this ovarian carcinoma cell line model (Anthoney et al, 1996, *Cancer Res*, **56**: 1374). Increased ONYX-015 replication is observed in three independent cisplatin-resistant derivatives of A2780. These cisplatin-resistant derivatives have loss expression of the hMLH1 mismatch repair gene. Restoration of hMLH1 gene expression by chromosome 3 transfer into a cisplatin-resistant derivative A2780/cp70 restores cisplatin sensitivity to near A2780 levels. However, the cells re-expressing hMLH1 are equivalently permissive for ONYX-015 replication as the parental hMLH1-deficient line and to chromosome 2 transfer controls. This lack of restoration of p53 function by hMLH1 re-expression is supported by lack of p53 transcriptional activity, as measured using expression-reporter constructs containing p53 consensus binding sites. The cells re-expressing hMLH1 do, however, regain a cisplatin-induced $G_2$ cell cycle arrest, suggesting that coupling of damage to $G_2$ arrest by hMLH1 may be a more important determinant of mismatch repair-associated cisplatin sensitivity than p53-mediated events in these cells.

### Up-regulation of the expression of hepatocyte growth factor activator (HGFA) in breast cancer

*S Hiscox, EL Davies and WG Jiang*

Department of Surgery, University of Wales College of Medicine, Heath Park, Cardiff, S. Wales, CF4 4XN, UK

Hepatocyte growth factor (HGF) is a pleiotropic growth factor that enhances the metastatic potential of breast cancer cells in vitro and thus may promote tumour growth and metastatic spread in vivo. HGF is secreted as an inactive single-chain form that is activated by the action of the serine protease HGFA in vivo.

The aim of this study was to examine the expression of HGFA in a series of breast cancer tissue pairs (tumour tissue and background normal tissue; number of pairs = 14). Total tissue protein was extracted from these tissues and subject to SDS-PAGE. Immunoprobing was then performed with anti-HGFA antibodies.

We observed the expression of a 34-kDa protein in all tissues; this protein corresponded to the active form of HGFA, produced from the inactive pro-HGFA by enzymic cleavage. This active HGFA protein was greatly up-regulated in 11 of 14 (78%) tumour tissues compared with their normal tissue counterparts. In 5 of 14 sample pairs (35%), a 97-kDa protein was detected corresponding to the inactive precursor form of HGFA (pro-HGFA). In each sample in which pro-HGFA was detected, expression was greater in the normal tissues than in their tumour counterparts.

We conclude that levels of active HGFA are elevated in breast tumour tissue, which may arise by the up-regulation of pro-HGFA to active-HGFA cleavage. Elevated levels of HGFA in tumour tissue may raise the levels of HGF itself within these tissues and thus promote tumour spread.

### A novel flow cytometric study of antigen expression to characterize cell type

*RL Loveday, J Greenman, MJ Kerin and JRT Monson*

Department of Surgery, Academic Surgical Unit, University of Hull, Cottingham Road, Hull HU6 7RX, UK

Characterization of cell types has traditionally been by immunohistochemistry, a non-quantitative technique. In this study, a novel flow cytometric technique has been used to allow analysis of surface and intracellular antigen expression in a series of colorectal and breast cancer cell lines. For analysis of intracellular markers, cells were fixed with a weak formaldehyde solution and then permeabilized with saponin. A panel of epithelial and fibroblast markers were assessed. These included BerEP4, EMA and 323A3 as cell surface epithelial markers and cytokeratin 19 as an intracellular epithelial marker. To identify fibroblasts, a surface fibroblast marker and intracellular vimentin were used. All of the cell lines showed strong staining for cytokeratin, whereas variable levels of the cell surface markers were found. As expected ZR751, T47D, MCF7, BT20, HT29 and LOVO showed strong staining for the three epithelial surface markers and cytokeratin and negative staining for the fibroblast marker and vimentin. In contrast, MCF7 ADR showed strong staining for both surface epithelial and fibroblast markers and also for vimentin and cytokeratin. LS180 showed better staining for 323A3 than the other epithelial surface markers, whereas CACO2 showed weak expression of all surface epithelial antigens.

© Cancer Research Campaign 1998

| | ZR751[a] | MCF7 ADR | LS180 | CACO2 |
|---|---|---|---|---|
| BerEP4 | 72[b] | 63 | 49 | 10 |
| EMA | 92 | 74 | 2 | 5 |
| 323A3 | 83 | 78 | 81 | 9 |
| Fibroblast | 19 | 84 | 6 | 42 |
| Vimentin | 11 | 90 | 7 | 86 |
| Cytokeratin | 92 | 90 | 71 | 88 |

[a]T47D, MCF7, BT20, HT29 and LOVO produced similar results to ZR751.
[b]These results are described as the percentage of positively stained cells.

The results indicate that the commonly used epithelial and fibroblast markers are not adequate in discerning cell populations; it appears that cytokeratin is the best marker for epithelial cells, however strong staining for cytokeratin does not exclude the presence of fibroblastic antigens.

In addition, when studying cell cycling, it was found that intracellular staining with MIB1 was far superior to Ki67 in assessing cell proliferation.

### Role of CD40 in anti-tumour immune responses

*AB Alexandroff, JA Ross and K James*

Department of Surgery, The University of Edinburgh, Lister Laboratories, RIE, Edinburgh EH3 9YW, UK

CD40 plays a crucial role in co-stimulation, survival and apoptosis of B and T cells and professional antigen-presenting cells. Despite being first identified on bladder carcinomas, very little is known about CD40 function in solid tumours. We examined the effect of CD40-ligand (CD40L) on the expression of cell-surface molecules and cytokine secretion by bladder and pancreatic carcinomas.

Pure CD40L trimer induced marked ICAM-1 and FAS but not MHC class II expression on CD40+ but not CD40– carcinomas cells. It also induced IL-6 but not IL-12 or IL-4 production. In control experiments, CD40L readily induced IL-12 in mononuclear cells obtained from healthy donors.

The intensity of CD40 expression could be enhanced but not induced by IFN-γ or patients' urine obtained after intravesical immunotherapy. No constitutive or induced expression of CD40L was observed on all cell lines studied. We have cloned (and sequenced) CD40 and CD40L into the retroviral vector pBabe.Puro. Currently, we are transfecting (CD40– CD40L–) γ carcinoma cell lines with CD40 and CD40L to establish whether this could (a) render them sensitive to CD40L; (b) enhance co-stimulation of lymphocytes reported by us previously.

ICAM-1 expression is essential for and correlates with cell-mediated killing of bladder cancer. FAS is essential for FAS ligand-mediated apoptosis of tumour cells. IL-6 is important for the generation of tumour-specific cytotoxic lymphocytes. Our results suggest that CD40–CD40L interactions may play an important role in the triggering and enhancing of effective anti-tumour responses.

This study was funded by the University of Edinburgh Faculty of Medicine Cancer Research fund. CD40L was kindly provided by Immunex. AB Alexandroff also acknowledges the receipt of a BACR travel fellowship.

### Cytogenetic determination of chromosomal aberrations in breast tumours

*RL Loveday, J Greenman, JRT Monson and MJ Kerin*

Department of Surgery, Academic Surgical Unit, University of Hull, Cottingham Road, Hull HU6 7RX, UK

Many gene mutations and chromosomal aberrations have been identified that are thought to be involved in malignant breast cancer. However, it is likely that many other genes, not yet identified, also have a role. Comparative genomic hybridization (CGH) allows the analysis of copy number changes across the entire tumour genome in a single hybridization. CGH is based on the competitive hybridization of two differentially labelled DNA samples to normal metaphase spreads. Tumour DNA is labelled with a green fluorochrome and normal reference DNA is labelled with a red fluorochrome. Image analysis compares the intensity of the green to red staining and calculates the ratio, thus allowing areas of amplification or deletion to be identified throughout the entire genome.

In this study, 15 consecutive breast tumours were analysed. All were staged according to standard TNM classification. Reference DNA was extracted from the patients' lymphocytes to provide a more sensitive reaction than using commercially available normal DNA, this is because heterochromatic regions are more comparable. To distinguish between somatic and germline mutations the tumour specimens were also compared with standard reference DNA (Vysis).

A wide variety of copy number changes were identified. Recurrent regions of amplification involved 1q (12 of 15), 3q (9 of 15), 8q (8 of 15), 12p (8 of 15) and 20p (9 of 15). The average number of amplifications per tumour was 12.7 ± 6.4, involving 8.5 ± 3.3 chromosomes in each tumour. Recurrent regions of deletion involved 17p (7 of 15) and 15p (7 of 15). The average number of deletions per chromosome was 10 ± 4.2, involving 5.1 ± 3.8 chromosomes in each tumour. The greatest number of aberrations (30) was found in a T4 tumour. The sites of most of the aberrations identified were distinct from those of currently known cancer genes, indicating that a number of other chromosomal loci are involved in the progression of malignant breast cancer. Further studies will be required to identify the genes and the role(s) they play in breast carcinogenesis.

### Evaluation of EGF receptor tyrosine kinase inhibitors identifies a potential selective inhibitor of MAP kinase

*J Edwards[1], CS Gibb[2], D Robins[2] and JMS Bartlett[1]*

[1]University Department of Surgery, Level II, Queen Elizabeth Building, Glasgow Royal Infirmary, Glasgow; [2]Department of Chemistry, University of Glasgow, Glasgow, UK

Epidermal growth factor (EGF) pathways have been shown to stimulate cellular proliferation in ovarian cancer cells, and EGF receptor expression is associated with poor prognosis in ovarian cancer patients. Therefore, we have investigated the possibility of using EGF-specific tyrosine kinase inhibitors as a novel treatment of ovarian cancer. We have screened a series of tyrosine kinase

© Cancer Research Campaign 1998

inhibitors, as potential therapeutic agents for the treatment of ovarian cancer. The potency of these drugs against EGF-stimulated PEO1 ovarian carcinoma cell growth was evaluated using an MTT assay (Table 1).

**Table 1** MTT results for EGF-mediated proliferation in PEO1 ovarian carcinoma cells

| Drug | $IC_{50}$ | Drug | $IC_{50}$ |
|---|---|---|---|
| CSG2 | 1.79 | CSG6 | 27.82 |
| CSG7 | 16.68 | CSG15 | 39.66 |
| CSG3 | 18.77 | CSG13 | 44.15 |
| CSG9 | 18.77 | CSG17 | 56.51 |
| CSG10 | 18.77 | CSG14 | 77.43 |
| CSG16 | 26.23 | CSG19 | 98.05 |

$IC_{50}$, dose causing 50% inhibition of growth factor-mediated proliferation over 6 days.

Of the 12 compounds evaluated using this system, CSG 2 was found to be the most potent inhibitor of EGF-stimulated cell growth. Further evaluation of CSG 2 and CSG 7 by Western blotting confirmed the ability of these agents to inhibit phosphorylation of the EGF receptor and its downstream targets. Activation of the EGF receptor kinase was inhibited by both CSG 2 and CSG 7 in a cell-free system where EGF receptor protein was extracted from A431 cells. EGF-stimulated MAP kinase activity was reversed when PEO1 cells were treated with CSG 2 or CSG 7. However, CSG 2 and CSG 7 were more active against activated MAP kinase ($IC_{50}$ value of 2 μM and 18 μM respectively) in a cell-free system than the EGF receptor ($IC_{50}$ value 26 μM and 59 μM respectively). This suggests that a principal site of action of both these drugs when inhibiting EGF-stimulated ovarian cell growth is at MAP kinase.

### Chromosome abberations in TCC predictive of disease outcome are independent events not associated with polyploidy

*JMS Bartlett[1], AD Watters[1], L Adie[1], JJ Going[2] and KM Grigor[3]*

University Department [1]of Surgery and [2]Pathology, GRI, Glasgow; [3]University Department of Pathology, Edinburgh, UK

Transitional cell carcinoma of the bladder (TCC) is the fourth commonest carcinoma in men, and frequent multiple recurrences (45% within 2 years) with risk of progression (10–20%) mandate extensive follow-up. Molecular markers of disease outcome should provide valuable diagnostic information allowing early treatment of patients at high risk of recurrence. We have previously reported that monosomy of chromosome 9 is predictive of recurrence, and polysomy of chromosomes 7 and 17 predicts progression in TCC. The current study was designed to establish whether such events were independent of tumour cell polyploidy.

Quantitative fluorescence in situ hybridization using alpha satellites was performed to detect chromosomes 10 and 11 in archival material from 12 patients with known status of chromosome 9, 7 and 17. Monosomy or polysomy was defined as a mean signal nuclear ratio (MSNR) outwith the normal range (1.5–1.8).

| Patient | Chrom 9 | Chrom 10 | Chrom 11 | Chrom 17 | Chrom 7 |
|---|---|---|---|---|---|
| NR1 | Monosomy | Disomy | Disomy | Disomy | Disomy |
| NR2 | Disomy | Disomy | Disomy | Disomy | Disomy |
| NR3 | Disomy | Disomy | Disomy | Disomy | Disomy |
| NR4 | Disomy | Disomy | Disomy | Disomy | Disomy |
| RNP1 | Monosomy | Disomy | Polysomy | Polysomy | Polysomy |
| RNP2 | Monosomy | Monosomy | Disomy | Disomy | Disomy |
| RNP3 | Disomy | Disomy | N.D. | Disomy | Disomy |
| RNP4 | Disomy | Disomy | Disomy | Polysomy | Polysomy |
| RP1 | Monosomy | Disomy | Disomy | Polysomy | Polysomy |
| RP2 | Monosomy | Disomy | Disomy | Disomy | Disomy |
| RP3 | Disomy | Disomy | Disomy | Disomy | Disomy |
| RP4 | Disomy | ND | ND | Polysomy | Polysomy |

ND, not done; NR, non-recurrer; RNP, recurrer non-progressor; RP, recurrer progressor. Disomy, 2*n*; monosomy, 1*n*; polysomy, 3+*N*.

In conclusion, loss of chromosome 10 occurs infrequently (9%), as does polysomy for chromosome 11 (10%). Polysomy of chromosomes 7 and 17 occurs independently of either monosomy 9 or 10 or, more significantly, polysomy 10 or 11. This finding would indicate that polysomy 7 and 17 identifies a chromosome-specific event unrelated to tumour polyploidy or monosomy 9.

### Liver resection for colorectal metastases: results and prognostic factors

*D Al-Musawi, P Hansen, H Tanaka, A Isla and NA Habib*

Department of Surgery, ICSM, Hammersmith Hospital, Du Cane Road, London W12 0NN, UK

From January 1990 until February 1997, 41 patients (12 male, 29 female, mean age 59 years, range 31–79 years) underwent 53 hepatic resections for liver metastases of colorectal primary tumours. Five, four and three patients underwent two, three and four resections, respectively, for recurrent liver metastases. The primary tumour was located in the rectum in 20 patients and the colon in the others. Liver metastases included 18 synchronous with the primary tumour, 23 metachronous, 23 unilobar and 18 bilobar. Major hepatic resection was performed in 34 (83%) patients and minor resection, which included segmentectomies and non-anatomical resection, in seven (17%) patients. Total vascular exclusion was used in 33 patients (mean ischaemic time 32 min) and the Pringle manoeuvre in eight patients (mean ischaemic time 39 min). There were 25 (61%) patients with 'potentially curative' initial liver resection and 16 (39%) patients with minimal macroscopic ($n$ = 12) or microscopic ($n$ = 4) residual disease. The mean (s.d.) operative blood transfusion was 2.1 (1.8) units packed red cells (range 0–8 units); 11 patients did not receive blood transfusion. Operative mortality was zero, but there was one in-hospital death 2 months after surgery (mortality rate 2.4%); non-fatal complications developed in 11 (27%) patients. Overall 2-year and 5-year survival rates after liver resection were 55% and 40%. Using Cox regression and log-rank tests: multiplicity of tumours, bilobar involvement, major or minor liver resection, synchronous or metachronous metastasis, sites of primary tumour, Dukes' staging and histological grading had no significant effect on survival. There were significant relationships with survival for size of tumour ($P$ = 0.0203), carcinoembryonic antigen ($P$ = 0.01) and preoperative alkaline phosphatase ($P$ = 0.04). Surgical resection is still the best available option for fit patients with resectable colorectal liver metastases.

 © Cancer Research Campaign 1998

### Genetic analysis of head and neck squamous cell carcinoma and surrounding uninvolved mucosa by comparative genomic hybridization

*JNE Ashman[1], J Greenman[2], SR Ell[1] and ND Stafford[1]*

[1]Department of Otolaryngology and Head and Neck Surgery, Hull Royal Infirmary, Anlaby Road, Hull; [2]Department of Surgery, Academic Surgical Unit, University of Hull, Cottingham Road, Hull HU6 7RX, UK

Squamous cell carcinoma of the head and neck has a high incidence of development of synchronous and metachronus primary tumours, relative to other solid tumours. Much interest has focused on the mucosa adjacent to primary tumours and led to the theory of 'field cancerization', in which prolonged exposure to carcinogenic insult leads to alteration of local mucosa.

Comparative genomic hybridization was used to investigate whether gene copy number aberrations could be detected in histologically normal mucosa from patients with squamous cell carcinoma. Samples were collected from 13 patients (nine laryngeal, three tonsil and one tongue carcinoma). Three tissue samples per patient were analysed: tumour, tumour margin (1 cm away) and distant 'normal' mucosa (5 cm from tumour). Genomic DNA extracted from each sample was nick labelled in the presence of fluorescein-12-dUTP and competitively hybridized against Spectrum-Red-labelled reference DNA. Analysis was performed on a Vysis Quips workstation to produce a copy number profile for each sample.

Copy number changes were not seen in genomic DNA extracted from any of the samples taken from 1 cm and 5 cm distant to the tumour. Common copy number changes were, however, seen in the tumour samples: deletions, 3p (9 of 13), 11q (6 of 13), 5q (5 of 13), 9p (5 of 13), 18q (5 of 13), 13q (5 of 13), 4q (4 of 13), 8p (4 of 13); amplifications, 3q (7 of 13), 8q (6 of 13), 5p (4 of 13), 11q (3 of 13). Simultaneous complete deletion of 3p and amplification of 3q occurred in 54% of tumours, suggesting the formation of the 3q isochromosome. Several loci of interest were identified containing known or putative oncogenes and tumour-suppressor genes involved in head and neck cancer progression. The region 3p24–p25.1 has previously been suggested as the site of a tumour-suppressor gene, and this was seen to be deleted in 70% of tumours. Amplification of the 11q13 locus occurred in 23% of the tumours. This region harbours the PRAD-1 gene coding for the cyclin D1 cell cycle-regulatory protein.

This study shows no evidence of copy number changes in the 'normal' mucosa of head and neck cancer patients, suggesting either that factors responsible for the putative 'field cancerization' are acting at a sub-chromosomal level or that the altered cells account for only a small proportion of the mucosa surrounding the tumour.

### Cytochrome P450 in human brain tumours

*MCE McFadyen[1], WT Melvin[2] and GI Murray[1]*

[1]Department of Pathology, [2]Department of Molecular and Cell Biology, University of Aberdeen, Foresterhill, Aberdeen AB25 2ZD, UK

The cytochromes P450 (P450) are a large group of constitutive and inducible oxidative enzymes with a major role in the metabolism of xenobiotics, including carcinogens and anti-cancer drugs. This family of enzymes are thus involved in tumour development and the response of established tumours to anti-cancer drugs. In this study, we have investigated the presence and cellular localization of individual families of P450 in normal human brain and primary brain tumours. Sections of formalin-fixed wax-embedded normal and tumour brain were used and the immunoreactivity of individual P450s was identified using light microscopic immunohistochemistry. In normal brain, immunoreactivity was observed for CYP1A, CYP2C, CYP2E1 and CYP3A, while there was no immunoreactivity for CYP1B1. Immunoreactivity in normal brain was mainly localized to neuronal cell bodies. All the tumours studied were astrocytomas, and CYP1A was present in 96% of tumours, while CYP1B1 was present in 87% of tumours. There was no significant immunoreactivity for CYP2C and CYP2E1. CYP3A immunoreactivity was identified in 43% of tumours. In all cases, positive immunoreactivity for each P450 was identified in tumour cells and, generally, in positively staining cells, the immunoreactivity was strong. The presence of individual forms of the CYP1 and CYP3A families in brain tumours provides further evidence for the expression of specific forms of P450 in malignant tumours. Individual forms of P450 in brain tumours could be important therapeutically for the development of anti-cancer drugs that are activated by those forms of P450.

This research was supported by grants from The Association for International Cancer Research and Tenovus-Scotland.

### Cytokine expression during ADCC of colorectal tumour cells using murine and humanized antibodies

*KM Gaskell, J Greenman, J Ashman, RJ O'Hara and JRT Monson*

Academic Surgical Unit, University of Hull, Hull HU6 7RX, UK

Antibody-dependent cell-mediated cytotoxicity (ADCC) is a reaction in which leucocytes bearing $F_c$ receptors recognize and lyse target cells, such as tumour cells, via specific antibodies. Little is known about the interaction between effector cells, antibodies and tumour cells, or about the mechanism of subsequent tumour cell lysis. In vitro and in vivo studies have consistently shown that humanized antibodies are vastly superior to murine antibodies in mediating ADCC. Therefore, expression of a panel of cytokines during an in vitro ADCC assay using both murine and humanized antibodies was investigated, to determine whether different types of antibody induce different cytokine mRNA expression. ADCC assays (18 h) were performed using the colorectal cell line HT-29 as targets and peripheral blood mononuclear cells from seven colorectal cancer patients and four normal donors as effector cells. The murine antibodies m17-1A and 323-A3 were tested, along with their respective humanized counterparts c17-1A and 3622W94. The irrelevant antibodies DB7/12 and 7E3 were also used as murine and humanized controls. Cytotoxicity was assessed using a colorimetric assay measuring lactate dehydrogenase (LDH) release from dead or damaged cells. Expression of various cytokines was assessed by RT-PCR using intron spanning oligonucleotide primers. The identity of PCR products was confirmed using restriction enzyme digestion.

The optimal antibody concentration and effector–target ratio were determined to be 1 μg $ml^{-1}$ and 50:1 respectively. Effector cells from patients with colorectal cancer mediated similar levels of ADCC to those from normal donors. In both the patient and the control groups, the humanized antibodies 3622W94 and c17-1A proved vastly superior in mediating ADCC to their murine counterparts, displaying levels of tumour cell lysis in the range of 80–90% and 40–50% respectively.

TNF-α, TGF-β, IL-1α, IL-1β, IL-6, IL-8, IL-10, IL-12 (p40) and IL-15 were consistently detected in ADCC conditions containing effector cells alone, both in the patient and in the control group. No additional cytokines were observed when effector cells were incubated with target cells and/or antibodies. Other control conditions using effector cells and antibody, or target cells and antibody, produced the same cytokine profiles as effector cells alone and target cells alone respectively. Varying the antibody concentration between 0.01 μg ml$^{-1}$ and 100 μg ml$^{-1}$ had no effect on the cytokine profiles. These findings indicate that, for the panel of cytokines tested, no cytokine mRNA is induced or 'switched off' during an 18-h ADCC reaction with either murine or humanized antibodies. However, there may be changes in the mRNA levels, but quantitative PCR analysis is required to investigate this.

### Characterization of a p53 oligomerization domain mutation isolated from a Li–Fraumeni-like family member

*ME Lomax[1], DM Barnes[2], SM Pickslev[3] and RS Camplejohn[1]*

[1]Richard Dimbleby Department of Cancer Research, UMDS, St Thomas' Hospital, London SE1 7EH; [2]ICRF Clinical Oncology Unit, Guy's Hospital, London SE1 9RT; [3]CRC Cell Transformation Group, Department of Biochemistry, University of Dundee, Dundee DD1 4HN, UK

The tumour-suppressor gene *p53* has been associated with the inherited cancer prone syndromes, Li–Fraumeni syndrome (LFS) and Li–Fraumeni-like syndrome (LFL). Recently Lomax et al (*Oncogene* 1997, **14**: 1869) described a point mutation in the oligomerization domain of *p53* (codon 337 R to C) in a LFL family member and Varley et al (*Oncogene* 1996, **12**: 2437) discovered a point mutation at codon 344 L to P, also in the oligomerization domain of *p53*, in a LFS family. Until recently, the role of point mutations in the oligomerization domain of *p53* was disputed.

The two described mutations have been generated in both *E. coli* and mammalian expression plasmids and the resulting proteins assessed for their abilities to perform certain functions of wild-type p53 protein. The oligomerization domain mutations have been compared with both wild-type p53 and with a core DNA binding domain mutation, codon 248 R to Q, which is a frequent mutation in human cancer.

The results obtained thus far suggest that the 344 L to P mutation is defective for DNA binding and induces apoptosis at levels about 20% of wild-type p53 and transactivates luciferase from a luciferase-*bax* reporter at about 6% of wild-type p53, in the p53-null cell line Saos-2. These results are very similar to those obtained with the 248 R to Q mutation. The 337 R to C mutation, however, does bind to DNA but is not fully functional at inducing apoptosis and transactivating luciferase from a luciferase-*bax* reporter, giving only 50–60% of wild-type activity. In addition, work on the inhibition of growth of Saos-2 cells by the various p53 mutants has also been carried out and a similar pattern of results has been found, in which wild-type p53 resulted in a very small number of foci, 344 L to P and 248 R to Q resulted in a large number of foci and 337 R to C resulted in an intermediary number of foci.

These results indicate that point mutations in the oligomerization domain of *p53* could play a role in tumorigenesis and also demonstrate the value of LFS and LFL families as models for the further understanding of the biological and biochemical properties of the *p53* tumour-suppressor gene.

### Genetic alterations of *N*-acetyl transferase in transitional cell carcinoma of the bladder

*AD Watters[1], MW Stacey[2], JJ Going[3], KM Grigor[4], TG Cooke[1], E Sim[2] and JMS Bartlett[1]*

[1]University Department of Surgery, GRI, Glasgow; [2]University Department of Pharmacology, Oxford; [3]University Department of Pathology, GRI, Glasgow; [4]University Department of Pathology, Edinburgh

Transitional cell carcinoma of the bladder (TCC) is the fourth commonest carcinoma in UK men and strong links with exposure to chemical carcinogens have been demonstrated [1,2]. *N*-acetyl transferase 1 and 2 are known to metabolize these compounds. The genes (*NAT1* and *2*) are located at 8p22 [3], and this region is frequently deleted in high-stage and -grade bladder tumours (detrusor muscle invasive) [4].

To further investigate a potential role for *NAT2* in TCC, a cohort of patients ($n$ = 19, no. of tumours 40) with previously diagnosed TCC and full clinical follow-up (mean 5 years) was studied. Dual-colour quantitative fluorescence in situ hybridization to detect *NAT2* and chromosome 8 was applied to archival material.

Three clear patterns of mutation were observed. Forty per cent (four of ten) of patients with non-invasive carcinoma showed a loss of *NAT2*, one of whom also had polysomy 8. Eighty-nine per cent (eight of nine) of patients with invasive carcinoma had aberrations of *NAT2*. Of these, 50% (four of eight) showed a loss of *NAT2*, three of whom also had polysomy 8. The remaining four patients demonstrated multiple copies of *NAT2*, possibly indicating gene amplification, and polysomy 8.

In conclusion, a large proportion of patients in this study had abnormalities of chromosome and gene copy number. *NAT2* was deleted in some cases while others had apparent increases in copy number. Selective retention of *NAT2* in some tumours has been reported [5], and the range of gene abnormalities may emphasize the complex metabolic role of *NAT2* in carcinogenesis.

### REFERENCES

1. Cartwright et al 1982 *Lancet* **2**: 842–846

2. Risch et al 1995 *Hum Mol Genet* **2**: 231–236

3. Hickman et al 1994 *Biochem J* **297**: 441–445

4. Knowles et al 1994 *Cancer Res* **54**: 531–538

5. Hubbard et al 1997 *Gut* **41**: 229–234

### Oral eicosapentaenoic acid reduces experimental human pancreatic tumour growth in SCID mice

*MD Barber, KCH Fearon and JA Ross*

University Department of Surgery, Royal Infirmary of Edinburgh, Edinburgh EH3 9YW, UK

We have recently shown that eicosapentaenoic acid (EPA), an $n$-3 fatty acid derived from fish oil, will attenuate cachexia in patients

© Cancer Research Campaign 1998

with advanced pancreatic cancer at a dose of 6 g daily with minimal side-effects (Barber et al, 1997, *Prostagland Leukot Essent Fatty Acids* **57**: 204). Others have suggested that EPA will slow experimental tumour growth in mice but most have used mixed fish oil preparations, administered the agent by non-physiological routes or given substantially more fat than is present in the normal diet. Many groups also began feeding experimental diets before tumour implantation. This study aimed to assess the effect of dietary high-purity EPA upon the growth of a human pancreatic cancer cell line in immunocompromised mice.

Fragments of tumour derived from the MIAPaCa-2 human pancreatic cancer cell line were transplanted subcutaneously on the left flank of 5- to 6-week-old female SCID (severe combined immunodeficiency) mice with Home Office approval. From the day after tumour implantation, mice were fed a sterile purified fat-free diet, based on American Institute of Nutrition guidelines, to which was added oil to make up 5% of the final diet weight using either corn oil or 95% pure EPA as free acid (4%, plus 1% corn oil). Mice were killed before the tumour burden became excessive at around 3 weeks. Groups of 12 mice on each diet were used concurrently on each occasion. Matched groups of animals without tumours were also studied.

Mice fed the diet enriched with EPA had significantly smaller tumours than control animals [median 2810 $mm^3$ (interquartile range 2050–3790) vs 4960 $mm^3$ (3210–6320), $P$ = 0.023, Mann–Whitney $U$-test]. Liver and spleens of tumour-bearing animals were substantially heavier than non-tumour-bearing controls ($P < 0.001$). Histological examination revealed multiple metastases in these organs in tumour-bearing animals. Liver and spleens of tumour-bearing animals were significantly lighter in those fed EPA compared with those fed corn oil ($P < 0.001$), suggesting a reduced metastatic burden.

Mice fed EPA ate significantly less food than controls (2.25 g $day^{-1}$ vs 3.05 g $day^{-1}$, $P = 0.021$). As diet restriction has been suggested to inhibit carcinogenesis, the experiment was repeated supplying matched amounts of food. Again tumours were significantly smaller in EPA-fed mice [3080 $mm^3$] (1800–3990) vs 4800 $mm^3$ (3060–6400), $P = 0.027$] and liver and spleens were significantly lighter with EPA ($P < 0.02$), suggesting that the tumour growth reduction was due to EPA and not diet restriction.

We conclude that 6–8 g $kg^{-1}$ EPA within the diet will slow the growth of a human experimental tumour and reduce metastatic spread in a mouse model. The potential of EPA as a non-toxic anti-cancer agent deserves further study in human neoplastic disease.

### Infectivity and selective replication of an E1B deficient adenovirus in mouse cell lines

*I Ganly[1], R Brown[1] and A Balmain[2]*

[1]CRC Department of Medical Oncology, CRC Beatson Laboratories, Glasgow G61 1BD, UK; [2]Onyx Pharmaceuticals, 3031 Research Dr. Bldg. A, Richmond, California 94806, USA

Selectively replicating adenoviruses may have a role to play in cancer therapy. At present, there is no immunocompetent mouse model to test replicating adenoviruses as previous work suggests that both the infectivity and the productive replication of adenoviruses in mouse cells is poor. The aim of this study was to develop an immunocompetent mouse model to test the activity of selectively replicating adenoviruses.

Infectivity of mouse cells from a wide range of tissue types was determined using a non-replicating E1A,E1B-deleted adenovirus with a lacZ reporter construct. Cell monolayers were infected at 0, 1, 10 and 100 p.f.u. $cell^{-1}$ and the percentage of beta galactosidase-positive cells measured 24 h post infection. Viral replication and cytolysis after wild-type adenoviral type 2 infection was determined by cytopathic effect assays and hexon protein staining at an infection titre of 10 p.f.u. $cell^{-1}$. Quantitation of replication was measured at 4 h and 72 h post infection by plaque-forming assays on 293 cell monolayers. The selectivity of a p53-dependent E1B-deleted adenovirus, Onyx-015, was tested on mouse epidermal cells of known p53 status using hexon protein staining and plaque-forming assays.

Contrary to previous work, infectivity of mouse cells of different tissue types was found to be high and was often greater than human cells at the same infection titre. The infectivity of 3T3 fibroblasts was, however, very low. Wild-type adenovirus Ad2 replicated in and produced a rising titre in specific mouse cell types, in particular mouse epidermal cells. However, the viral yield was 10–50 times less than in human cells. Onyx-015 showed selective replication in mouse epidermal cells that lacked functional p53.

Adenoviral infectivity of mouse cells is high and productive infections will occur in specific tissue types notably epidermal cells. Using Onyx-015 virus, replication is shown to be selective for p53 mutant cells. These studies will enable us to test the activity of oncolytic adenoviruses in vivo in immunocompetent mice.

### Virus-directed enzyme prodrug therapy using retrovirally delivered *E. coli* nitroreductase and CB1954

*IA McNeish[1], MG Gilligan[1] NK Green[2], SM Roberts[1], DJ Kerr[1], F Friedlos[3], CJ Springer and PF Searle[1]*

[1]CRC Institute for Cancer Studies and [2]Department of Surgery, University of Birmingham, B15 2TA; [3]Institute for Cancer Research, Sutton, SMG 5NG, UK

The *E. coli* enzyme nitroreductase (NTR) converts the weak, monofunctional alkylating agent CB1954 (5-(aziridin-1-yl)-2,4-dinitrobenzamide) into a highly active bifunctional metabolite that is toxic to both cycling and non-cycling cells. Using high-density cell mixing experiments with a SKOV3 clone expressing high-level NTR, we have previously shown that a significant bystander effect exists in vitro for the NTR/CB1954 combination: when only 5% of cells express NTR, the $LD_{50}$ is reduced tenfold compared with parental cells and, if 30% of cells in the mixture express NTR, the population acts as if 100% are NTR positive.

We have used the packaging cell lines gp+env AM12 and FLY RD18 to produce the recombinant retrovirus LNC-ntr at titres of 1–5 × $10^5$ c.f.u $ml^{-1}$. Using this virus, we have transduced the ovarian carcinoma lines SKOV3, IGROV1 and cisplatin-resistant A2780 CP, as well as early-passage cells derived from the ascites of patients with advanced ovarian carcinoma. Single transduction gives an MOI of approximately 1 and a transduction efficiency of around 25%, and produces a tenfold increase in sensitivity to CB1954 of bulk unselected cells without reliance on antibiotic selection or cell cloning procedures.

One of the main disadvantages of retroviruses as vectors for gene therapy is their relatively low titre. Using low-speed (6000 ***g***) overnight centrifugation, we have been able to increase retroviral

© Cancer Research Campaign 1998

titre 100-fold to approximately $5 \times 10^7$ c.f.u. $ml^{-1}$. Using this concentrated virus, we have transduced SKOV3 and A2780 cells with transduction efficiencies approaching 100% to produce a 400-fold increase in bulk unselected cell sensitivity to CB1954.

To improve the ability to visualize NTR expression in vitro and in vivo, we have tagged NTR with a novel 30mer derived from the Human Papilloma Virus Type 1 E4 gene. We have shown that the tagged NTR is still functional in vitro and can be detected by anti-HPV E4 monoclonal antibodies.

We have grown subcutaneous tumours in nude Balb C mice using SKOV3 cells and a SKOV3 clone expressing high-level NTR. Two intraperitoneal injections of CB1954 (50 mg $kg^{-1}$) given 1 week apart produce marked growth delay of the NTR tumours, although not complete cure, and a corresponding increase in survival in these animals.

### HPMA copolymer platinates as novel anti-cancer agents

*E Gianasi[1], E Evagorou[1], RG Buckley[1], S Kneller[1], N Malik[1], G Wilson[2] and R Duncan[1]*

[1]Centre for Polymer Therapeutics, School of Pharmacy, London WC1N 1AX; [2]Access Pharmaceuticals, Dallas, USA

Since its discovery cisplatin has had a major impact on cancer chemotherapy, but new platinum analogues have shown minimal improvement compared with cisplatin. Preparation of polymer platinates is an attractive strategy to reduce systemic toxicity, to localize more drug in the tumour via the enhanced permeability and retention (EPR) effect and to overcome resistance. The concept of polymeric anti-cancer agents (Duncan et al, 1996, *STP Pharma Sci* **6**: 237) has been established clinically in the form of the *N*-(2-hydroxypropyl) methacrylamide (HPMA) copolymer doxorubicin (PK1) (Vasey et al, 1996, *Ann Oncol* **7**: 97). The design of effective polymer platinates is challenging. It is essential to select a biocompatible carrier and to release a biologically active platinate in the tumour, and that the platinum is tightly bound to the polymer during transport in the circulation and during elimination of the conjugate via the kidney.

Four classes of polymer have been investigated as polymeric platinates: natural polymers, poly(amidoamine)s, PAMAM dendrimers and HPMA copolymers, with the last being selected for development of a clinical candidate. HPMA conjugates ($M_r$ ~ 30 000; Pt content 5–10 wt%) were prepared containing pendant peptidyl side-chains terminating in ligands capable of coordinating platinum. Active Pt species are released from the conjuate either by hydrolysis or by enzymatic degradation of the peptidyl side-chains. Release rates have been tailored by conjugate design. In vivo evaluation against a panel of tumours has shown that several conjugates display equi-effective anti-tumour activity compared with cisplatin after i.p. administration to treat an i.p. tumour, thus confirming their ability to liberate active Pt species in vivo. However, when administered i.v. to treat s.c. B16F10 melanoma, the polymeric conjugates showed a dramatic increase in activity. Pharmacokinetic studies have shown that Pt conjugation leads to an increase in B16F10 AUC of 63-fold compared with that achieved at the MTD for cisplatin in this model. This is consistent with polymeric tumour capture by the EPR effect. The polymeric platinates were less toxic than cisplatin (two- to 15-fold) and warrant clinical evaluation.

### Inhibition of angiogenic factor- and tumour-induced angiogenesis by gamma linolenic acid

*J Cai, WG Jiang and RE Mansel*

University Department of Surgery, University of Wales College of Medicine, Cardiff, UK

Angiogenesis is an essential event in the formation of metastasis. We examined the effect of gamma linolenic acid (GLA), an anti-cancer polyunsaturated fatty acid, on the angiogenesis and motility of vascular endothelial cells.

A rat aortic ring assay was used to determine angiogenesis [spontaneous, angiogenic factor or tumour cell (MDA MB 231 breast cancer) induced]. The motility of a human vascular endothelial cell line, ECV304, was assessed using a cytocarrier assay and colloidal gold phagokinetic assay. GLA was tested over a range of concentrations (1–50 μM). In a three-dimensional matrix, aortic rings generated new vessels after 3 days, reaching a maximum number after 14 days. The angiogenesis was stimulated by both angiogenic factor (scatter factor) (number of new vessels ± s.e.m. was 15.0 ± 3.2) and breast cancer cells (33.6 ± 3.5), compared with control (11.0 ± 2.4). Inclusion of GLA in this assay resulted in a concentration-dependent inhibition of angiogenesis in non-stimulated (0.4 ± 0.3), angiogenic factor-stimulated (2.0 ± 0.8) and tumour cell-stimulated (12.7 ± 4.3) cultures. In the motility assay, inclusion of GLA reduced the migration of the endothelial cells (ECV304) as shown by a reduction in phagokinetic clearance of colloidal gold particles. In the cytocarrier motility assay, a significant reduction of the motility of ECV304 by gamma-linolenic acid was also seen (motility index being 53.3 ± 4.5 with GLA vs 85.0 ± 11.7 in control, $P = 0.011$ by Student's *t*-test).

We conclude that gamma linolenic acid, a *n*-6 series essential fatty acid, inhibits angiogenic factor- and tumour cell-induced angiogenesis in vitro. This inhibitory effect is partly due to its inhibition of the motility of vascular endothelial cells.

### Evidence for an immunomodulatory role for α-MSH in cutaneous and ocular melanoma

*JW Haycock[1], K Dorset[1], M Wagner[1], IR Rennie[2] and S MacNeil[1]*

Departments of [1]Medicine and [2]Ophthalmology and Orthoptics, Clinical Sciences Centre, Northern General Hospital, Sheffield S5 7AU, UK

Normal human melanocytes may require α-MSH to resist the action of ultraviolet (UV) light, which has the potential to initiate cellular damage and local inflammation. Recently, we have also found α-MSH to oppose the ability of TNF-α to up-regulate ICAM-1, leading us to speculate that α-MSH may have a role in down-regulating particular cell-surface molecules necessary for immune system recognition and targeting (e.g. ICAM-1), which are normally inducible via inflammatory cytokines (e.g. TNF-α). We investigated this concept by considering how α-MSH might oppose the ability of TNF-α to activate relevant transcription factors in human cutaneous and ocular melanoma cells.

© Cancer Research Campaign 1998

NF-κB is a transcription factor responsible for expression of several genes, many of which are necessary for a successful response to cellular stress. It is known to be inducible by a number of cellular agents (e.g. cytokines) and also environmental factors (UV light). We studied the functional DNA binding activity of NF-κB using electrophoretic mobility shift analysis in A375-SM human cutaneous melanoma cells. Cells showed little constitutive expression of the p50:p65 heterodimer NF-κB complex. However, incubation with TNF-α (200 U $ml^{-1}$) for 2 h gave a maximum level of up-regulated activity in this particular complex alone. Importantly, co-incubation of the cells with TNF-α (200 U $ml^{-1}$) and α-MSH ($10^{-10}$ M – $10^{-8}$ M) gave a significant decrease in the level of the TNF-α-induced NF-κB activity (by approximately 50%, over 2 h). [α-MSH alone ($10^{-10}$ M – $10^{-8}$ M) had no effect on functional binding activity.] In addition, the co-incubation of cells with TNF-α (200 U $ml^{-1}$) and IBMX (1 mM) for 2 h also showed a 50% decrease in the level of TNF-α inducible activity, suggesting that the action of α-MSH can be mimicked by a receptor-independent elevation in cAMP. In contrast, analysis of an ocular melanoma cell line revealed a high constitutive activity of NF-κB that was unresponsive to the action of TNF-α or α-MSH. However, an alternative Rel family complex (possibly p50) was upregulated by TNF-α (200 U $ml^{-1}$, 1 h), and this increase was reduced by co-incubation of the cells with TNF-α and α-MSH ($10^{-9}$ M). These results support the hypothesis that α-MSH may be acting as an immunomodulatory peptide in suppressing the response of melanoma cells to cytokines, which might otherwise bring these cells to the attention of the immune system.

### HGF/SF induces disruption of intercellular tumour cell adhesion: involvement of β-catenin

*S Hiscox and WG Jiang*

Department of Surgery, University of Wales College of Medicine, Cardiff, S. Wales, CF4 4XN, UK

Metastasis is the single, most important factor that determines cancer patient prognosis. Early stages of epithelial tumour cell metastatic spread involve loss of E-cadherin-mediated cell–cell adhesion within the primary tumour mass. Hepatocyte growth factor/scatter factor (HGF/SF) greatly stimulates the metastatic potential of tumour cells and promotes the 'scattering' of tightly packed tumour cell colonies. The aims of this study were thus to investigate the effects of HGF/SF on E-cadherin (E-cad) and its associated catenin molecules (α, β and γ) in epithelial tumour cells.

The effects of HGF/SF on two colorectal carcinoma cell lines, HT115 (E-cad⁻) and HRT18 (E-cad⁺), were monitored by time-lapse video microscopy. The expression and tyrosine phosphorylation of E-cadherin and catenins in response to HGF/SF stimulation were determined by immunocytochemistry and Western blotting/immunoprecipitation respectively.

HRT18 cells were observed to form very tight colonies in culture, while HT115 cells formed colonies in which cells were loosely packed. HGF/SF caused significant scattering of HT115 cell colonies and promoted the disruption of intercellular cell–cell adhesions in HRT18 cell colonies. This resulted in the partial scattering of these colonies. HGF/SF had no effect on the overall level of E-cad or associated catenin molecules in HRT18 cells, but did enhance the tyrosine phosphorylation status of β-catenin in these cells, an effect that was observed as early as 30 min after HGF/SF treatment. The amount of β-catenin co-precipitating with E-cad was reduced after HGF/SF treatment.

We conclude that HGF/SF-induced phosphorylation of β-catenin results in a reduction of association of this molecule with E-cadherin and thus a loss of function of the E-cadherin adhesion complex. The association of catenin molecules with cell-surface cadherin is an essential requirement for cadherin-mediated cell–cell adhesion. These data therefore implicate HGF/SF as a possible regulator of early events in the metastatic progression of tumours by promoting loss of E-cadherin-mediated cell–cell adhesion.

### Inhibition of colon tumour promoter-induced COX2 mRNA induction by curcumin correlates with inhibition of NFkB DNA-binding and transactivation

*KA Holloway[1], RJL Munks[2], M-A Peer[1] and SM Plummer[1]*

[1]Centre for Mechanisms of Human Toxicity and [2]MRC Toxicology Unit, University of Leicester, Leicester LE1 9HN, UK

Prostaglandins may play an important role in colon carcinogenesis. Non-steroidal anti-inflammatory agents (NSAIDs), which inhibit their production by prostaglandin H synthase (PGHS), may reduce colon cancer risk by 50% and can inhibit development of colon tumours in animal models of this disease. The COX2 gene, an inducible form of PGHS, is overexpressed in human colon tumours and is a potential target for the chemopreventive effects of these agents. In this study, we have investigated the effects of endogenous colon tumour promoters, fecapentaene-12 (fec-12) and TNF-α on COX2 gene expression in the human colon epithelial cell line HCEC and human colon carcinoma cell line SW480. Furthermore, we have investigated the mechanism of action of the colon cancer chemopreventive agent curcumin (CUR). Exposure of HCEC cells to TNF-α (0.1–10 ng $ml^{-1}$) or fec-12 (10–40 μM) for 2 h caused a dose-dependent two- to sevenfold induction of COX2 mRNA levels, measured by RT-PCR. The TNF-α and fec-12 induction of COX2 mRNA peaked at 3 h and returned to control levels by 24 h. As there is evidence that COX2 gene induction is controlled in part by the transcription factor NFkB, we also investigated the effects of these agents on NFkB-DNA binding to 'consensus' or 'COX2-promoter' NFkB oligonucleotides by EMSA. TNF-α (0.1 or 10 ng $ml^{-1}$) and fec-12 (20 μM) caused a four- to five- and fourfold induction of NFkB DNA binding to these oligos respectively. Treatment of SW480 cells with TNF-α caused a threefold induction of luciferase expression in transfection studies with a luciferase reporter construct containing six NFkB binding sites (pNFkB). Pretreatment of cells with CUR (10–40 μM) for 1 h before TNF-α or fec-12 exposure caused a dose-dependent 20–90% inhibition of COX2 mRNA induction. CUR caused a dose-dependent 30–90% inhibition of NFkB DNA-binding activity in EMSA assays and inhibited the activation of pNFkB-mediated luciferase activity in reporter construct transfection experiments with the colon tumour cell line SW480. These results indicate that the colon cancer chemopreventive effects of CUR may be mediated in part through down-regulation of COX2 gene expression through inhibition of NFkB activation.

© Cancer Research Campaign 1998

### Cancer in the parents of children with medulloblastoma

*V Blair, JM Birch, AM Kelsey and M Newbould*

CRC Paediatric & Familial Cancer Research Group, Royal Manchester Children's Hospital, Manchester M27 4HA, UK

Medulloblastoma occurs in families with genetic syndromes, such as Li–Fraumeni (LFS) and Gorlin's syndrome (GS). To estimate cancer risks in parents of children diagnosed with medulloblastoma before age 15 years and the proportion of paediatric medulloblastoma cases that might have a genetic component, 167 cases diagnosed from 1954 to 1993 and registered with the Manchester Children's Tumour Registry were identified. A total of 320 parents were traced and flagged. Numbers of cancers expected in the parents in the period from the latest of 1 January 1965 and the birth of the case to the earliest of 31 December 1993; date of last follow-up and 75th birthday were calculated from population rates for the NW of England and compared with observed numbers, which were assumed to follow a Poisson distribution. Two-sided hypothesis tests were performed to determine whether observed numbers differed significantly from those expected. Overall, 21 cancers were observed and 21.86 expected (RR = 0.96, 95% CI 0.59–1.47, $P = 0.97$). Eight breast cancers occurred in mothers, but only 3.33 were expected (RR = 2.4, 95% CI 1.04–4.73, $P = 0.04$). The excess of breast cancers was significant for mothers of cases diagnosed under the median age (7 years) at onset (0 = 6, E = 1.75, RR = 3.43, 95% CI 1.26–7.46, $P = 0.02$) and of women (0 = 4, E = 1.09, RR = 3.65, 95% CI 1.00–9.36, $P = 0.05$), but not for mothers of cases diagnosed aged 7 and above (0 = 2, E = 1.57, RR = 1.27, 95% CI 0.15–4.59, $P = 0.93$) or of men (0 = 4, E = 2.23, RR = 1.79, 95% CI 0.49–4.59, $P = 0.37$). No other cancers occurred to excess but, overall, a significant deficit occurred in fathers that could not be accounted for by underascertainments (0 = 3, E = 11.6, RR = 0.26, 95% CI 0.05–0.76, $P = 0.006$). Twenty-three families in which a parent was diagnosed with cancer were interviewed or completed a questionnaire. Inspection of the pedigrees identified the following families: one LFS, one Li–Fraumeni-like, two GS, one FAP and five with potentially interesting clusters of cancers. The percentage of medulloblastoma cases identified by this method to be associated with a genetic component was 3–6%.

### Seasonal variations in the onset of childhood leukaemia and lymphoma

*RMC Westerbeek, OB Eden, V Blair, AM Kelsey and JM Birch*

CRC Paediatric & Familial Cancer Research Group, Royal Manchester Children's Hospital, Manchester M27 4HA, UK

Infection is suspected to be a factor in the aetiology of leukaemia. If seasonal variation in onset of leukaemia or lymphoma could be demonstrated, this would provide support for a link with exposure to infection. Therefore, all cases in the Manchester Children's Tumour Registry diagnosed with acute lymphoblastic leukaemia (ALL), acute non-lymphocytic leukaemia (ANLL), Hodgkin's disease (HD) or non-Hodgkin lymphoma (NHL) between 1 January 1954 and 31 December 1996 were included in an analysis of seasonal variation in the month of first symptom and the month of diagnosis. Cases of common ALL (c-ALL) diagnosed from 1979 onwards were also analysed separately. The groups considered for analysis were all cases of ALL ($n = 1070$), ALL diagnosed under 18 months of age ($n = 74$), ALL diagnosed between 18 and 95 months ($n = 730$), ALL diagnosed over 95 months ($n = 266$), c-ALL ($n = 309$), ANLL ($n = 244$) HD ($n = 166$) and NHL ($n = 189$). Data analysis included chi-squared test for heterogeneity to look for departure from a uniform distribution throughout the year and Edward's test to detect cyclical trends (sinusoidal curve of period 12 months) over the year. Tests for heterogeneity based on date of first symptom were significant for HD ($P = 0.002$) and of borderline significance for ALL aged 0–179 months ($P = 0.055$) and c-ALL ($P = 0.056$). Tests for cyclical trends based on date of first symptom were significant for c-ALL ($P = 0.037$), with peak month November, and HD ($P = 0.001$), with peak month December, and of borderline significance for ALL aged 0–179 months ($P = 0.07$), with peak month March. No evidence of seasonal variation was found in other diagnostic groups for date of first symptom or in any diagnostic group for date of diagnosis. These results provide supportive evidence for an infectious aetiology in c-ALL and HD. Date of first symptom may reflect actual biological onset more accurately than date of diagnosis.

*© Cancer Research Campaign 1998*