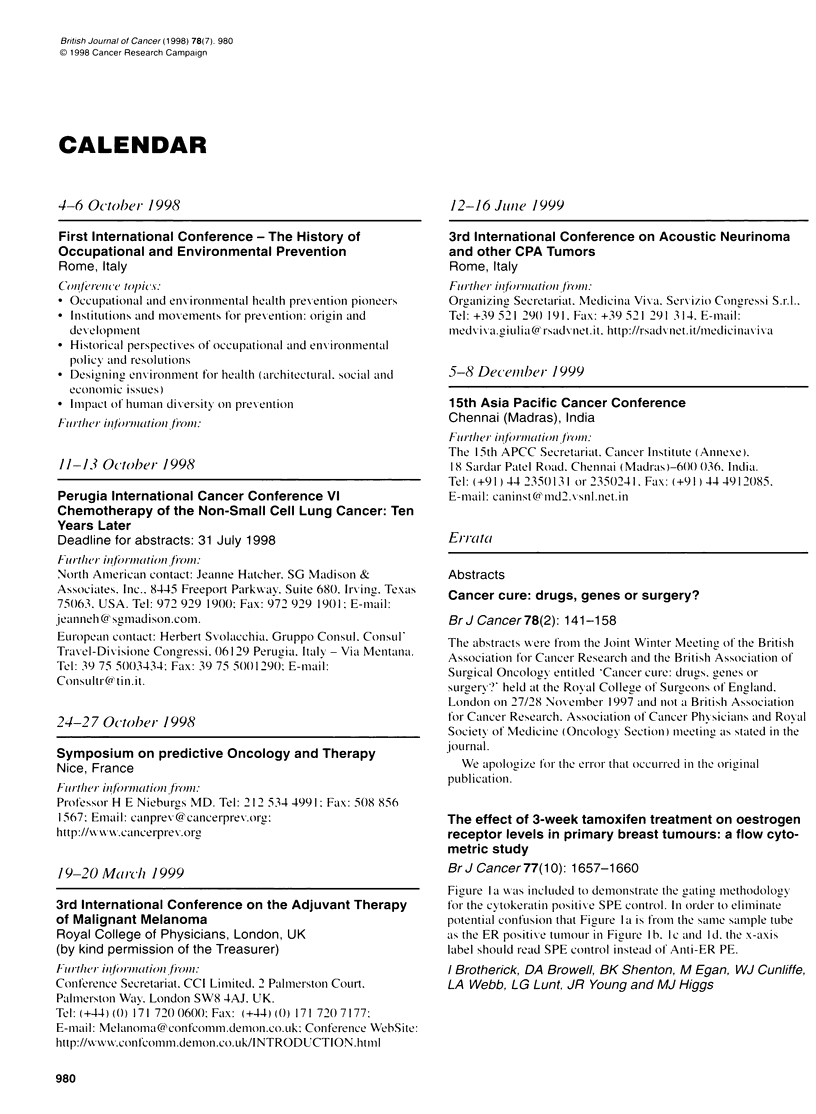# Cancer cure: drugs, genes or surgery?

**Published:** 1998-10

**Authors:** 


### *Errata*

Abstracts

### Cancer cure: drugs, genes or surgery?

*Br J Cancer* **78**(2): 141–158

The abstracts were from the Joint Winter Meeting of the British Association for Cancer Research and the British Association of Surgical Oncology entitled 'Cancer cure: drugs, genes or surgery?' held at the Royal College of Surgeons of England, London on 27/28 November 1997 and not a British Association for Cancer Research, Association of Cancer Physicians and Royal Society of Medicine (Oncology Section) meeting as stated in the journal.

We apologize for the error that occurred in the original publication.